# Reconstructing the geological provenance and long-distance movement of rectangular, fishtail, and *croisette* copper ingots in Iron Age Zambia and Zimbabwe

**DOI:** 10.1371/journal.pone.0282660

**Published:** 2023-03-22

**Authors:** Jay Stephens, David Killick, Shadreck Chirikure

**Affiliations:** 1 School of Anthropology, University of Arizona, Tucson, AZ, United States of America; 2 Archaeometry Laboratory, Research Reactor Center, University of Missouri, Columbia, MO, United States of America; 3 School of Archaeology, University of Oxford, Oxford, United Kingdom; 4 Department of Archaeology, University of Cape Town, Cape Town, South Africa; Istanbul University: Istanbul Universitesi, TURKEY

## Abstract

The southern third of Africa is unusually rich in copper ore deposits. These were exploited by precolonial populations to manufacture wound-wire bangles, other forms of jewelry, and large copper ingots that were used as stores of copper or as forms of prestige. Rectangular, fishtail, and *croisette* ingots dating between the 5^th^ and 20^th^ centuries CE have been found in many locations in the Democratic Republic of the Congo (DRC), Zambia, and Zimbabwe, with isolated finds in Malawi and Mozambique. Molds for casting these ingots have been found mostly in the Central African Copperbelt, but also around the Magondi Belt copper deposits in northern Zimbabwe. For years, scholars have debated whether these ingots were exclusively made in the Copperbelt or if the molds found in Zimbabwe indicate that local copies were produced from Magondi Belt copper ore (Garlake 1970; Bisson 1976). Before the recent application of lead isotopic and chemical methods to provenance copper in central and southern Africa, there was no way to discern between these hypotheses. Rademakers et al. (2019) and Stephens et al. (2020) showed that copper artifacts from southern DRC (mostly from Upemba) and from northwestern Botswana (Tsodilo Hills) match the lead isotope ratios of ores from the Copperbelt. Building upon these previous studies, we present here the first results from a copper provenance project across the southern third of Africa, from the Copperbelt to northern South Africa. We apply lead isotopic analysis (LIA) and chemical analyses to establish the provenance of 29 *croisette* ingots recovered in Zimbabwe, 2 fishtail and 1 rectangular ingot recovered from sites in Zambia, and an “X” shaped ingot smelted in an experiment in Zambia in the 1970’s. Our chemistry and lead isotopic results indicate that 16 of these objects were smelted with copper from the Copperbelt, 16 objects source more specifically to the Kipushi deposit within this geological district, and only one HXR ingot sources to the Magondi Belt in Zimbabwe. Taken together, we clearly illustrate that *croisette* ingots were traveling significant distances to reach their eventual sites of deposition, and that there was also local production of these objects in Zimbabwe.

## Introduction

**Fig 1:**
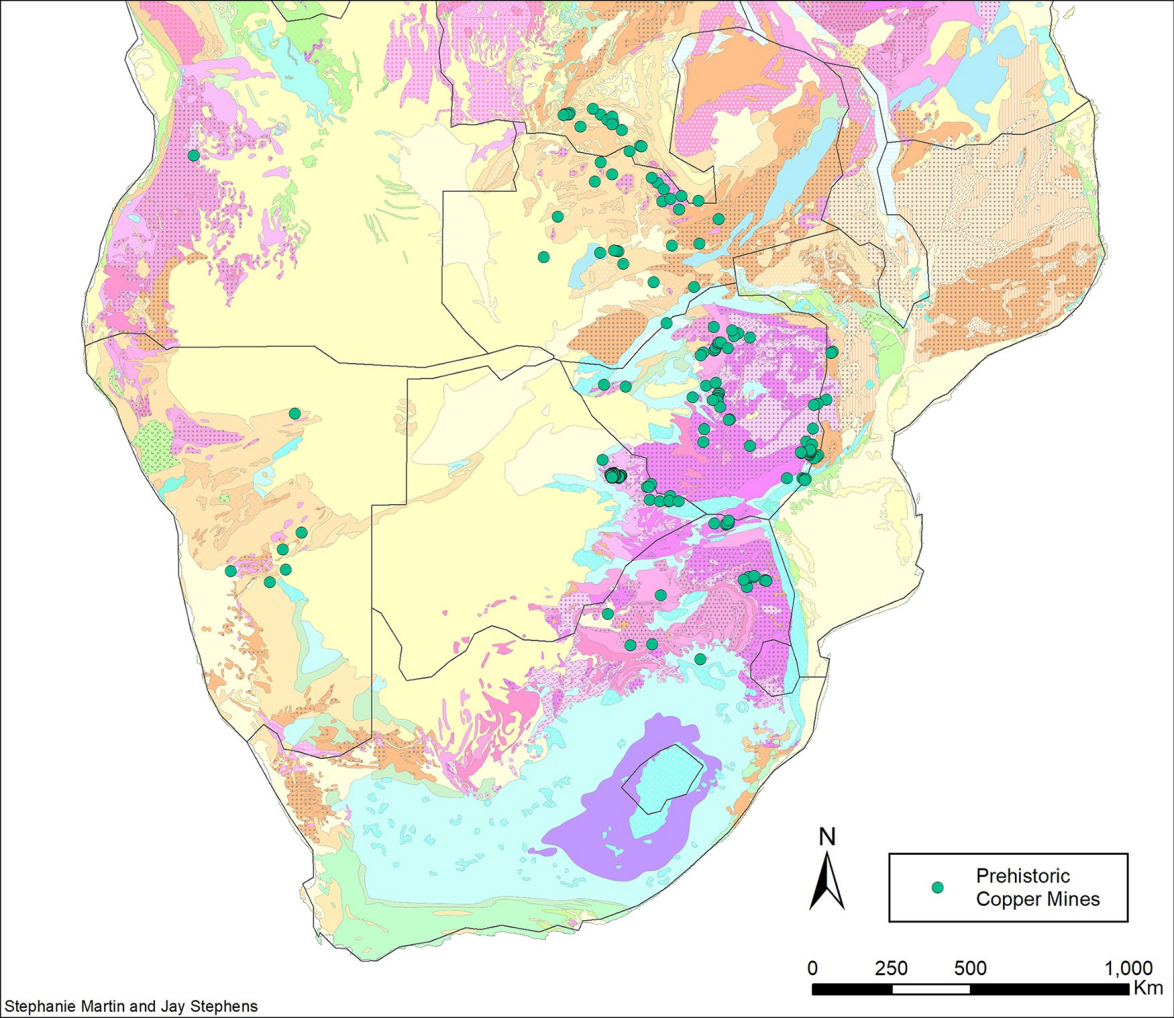
Locations of prehistorically exploited mines in southern Africa. For location and discussion of these mines, see Bisson [2], Chirikure [10], Evers and van der Berg [5], Friede [11], Hammel et al. [12], Herbert [1], Huffman et al. [13], Killick et al. [14], Mason [15], Miller [16], Miller and Sandelowsky [17], Molyneux and Reinecke [18], Phimster [19], Summers [6], Swan [8], Van Waarden [7]. For a geological legend to the map, see **Fig C in the S1 Appendix**. (Geological basemap adapted from Thiéblemont et al. [20]).

The southern third of Africa—from the southernmost Democratic Republic of Congo (DRC) to South Africa—is rich in copper ore deposits (**Fig 1**). These include the sediment-hosted deposits in the Central African Copperbelt (draped along the border between DRC and Zambia), deposits of similar type in the Magondi Belt of northern Zimbabwe, the early Precambrian metavolcanic “greenstone belts” of southern Zimbabwe, eastern Botswana and northeastern South Africa, the Limpopo Mobile Belt (mostly gneisses) parallel to the border between Zimbabwe and South Africa, and the Phalaborwa Igneous Complex (a carbonatite) in northeastern South Africa. All of these were extensively mined before 1500 CE, when Europeans first reached southern Africa [1]. Several significant studies of copper mining were undertaken between 1920 and 1975 [1–7], but there have been few archaeological investigations of mines since then [8, 9]. Archaeological evidence at and around these mines is disappearing rapidly as a result of modern mining ventures, particularly in the Copperbelt area.

**Fig 2:**
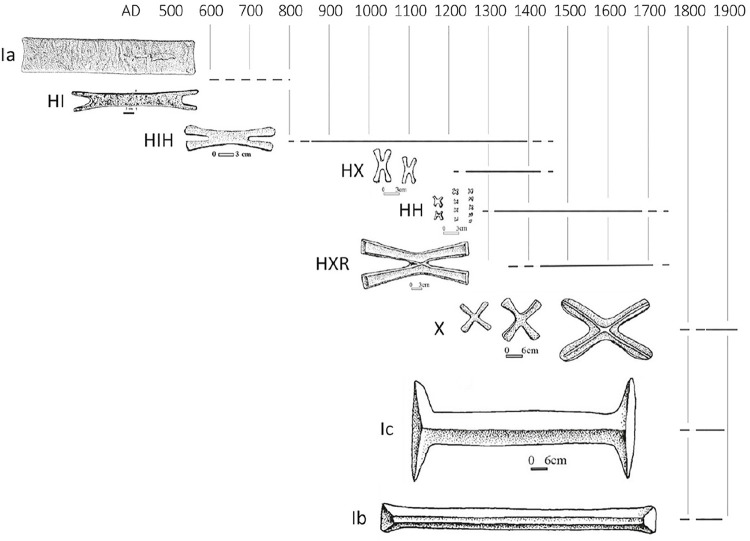
“Ia” (rectangular and fishtail) and *croisette* ingot typology. (Reproduced from Rademakers et al. [24], Fig 2A).

On present evidence, the earliest date for the exploitation of copper minerals in southern Africa is the 4^th^ century cal CE at Kansanshi mine in Zambia [2], agreeing with the timing for the arrival of Bantu agriculturalists into the region [21]. Miners at these deposits used iron tools, hammerstones, and fire-setting to extract ore minerals [6, 10]. Copper minerals were either smelted near the mine, as at Kansanshi, or transported elsewhere to be reduced to copper metal, as at Kipushi where smelting sites were located on the banks of the Kafue river, tens of km from the mines [2]. Documented furnaces in the Copperbelt and further to the south show that copper was 1) tapped directly into molds, 2) allowed to solidify at the furnace bottom, or 3) later recovered and refined from prills trapped in slag [10]. It could then be worked into various forms using tools like hammers and the iron wire-drawing plates excavated from Ingombe Ilede [2, 10, 22]. Copper in the archaeological record of southern Africa typically appears in the form of wound-wire bangles or other jewelry, but also includes large copper ingots that were used as stores of copper, forms of prestige, and/or circulated as general or limited purpose currency [23]. The distribution, dates, and uses of these large ingots (**Fig 2**) have been well studied, however the provenance of their copper has yet to be resolved. This paper focuses on the provenance of a subset of these ingots, and our isotopic and chemical results identify three geological sources: the Central African Copperbelt, the Kipushi deposit within the Copperbelt, and the Magondi Belt.

### Geological overview

#### Central African Copperbelt

The precise age of copper mineralization in the Copperbelt remains sharply debated (see discussion between Hitzman and Broughton [25] and Sillitoe et al. [26]). Copperbelt copper deposits are hosted in sediments that collected between the Zimbabwean and Congo Cratons prior to the assembly of Gondwana between 650 and 500Ma [27]. Later compressional forces related to the closure of this marine basin overthrust, faulted, and folded these sedimentary sequences to form the Damaran-Lufilian Arc, which runs along the coast from western South Africa, through Namibia, and up into Zambia and the southern DRC. One current metallogenic model is that main-stage mineralization may have occurred during sediment diagenesis prior to the Lufilian (Pan-African) orogeny. The other model holds that it occurred during the orogeny. Dates from Selley et al. [28, Appendix] also suggest that each of these episodes may have formed some mineralization. Whatever the timing of regional metallogenesis, the Lufilian episode almost certainly remobilized and redistributed existing mineralization [29]. The geological record also includes two uranium influx episodes at 650Ma and 530Ma [30], which contributed excess ^206^Pb and ^207^Pb to ore minerals via radioactive decay of ^238^U and ^235^U and produced broad isotopic overlap between most deposits in the Copperbelt.

Most of the Copperbelt deposits are stratiform Cu-Co(-U) deposits, with varying concentrations of Ni, U, Ag, Au, PGE, Se, Mo, V, Te, As, and Th. They are often depleted in Pb and other chalcophile elements, which occur in much higher levels in younger epigenetic vein-type deposits at Kipushi [30, 31]. Cu-Co deposits in the Copperbelt are also famous for their extremely pure malachite deposits, which fill voids and form stalactites in dolomitic karsts [32]. These large pieces of pure malachite can be smelted without fluxing agents or the formation of a slag [24, 33–35].

#### Kipushi

Although Kipushi is within the Copperbelt in a geographical sense, it is geologically quite different from other Copperbelt deposits. It is younger than other deposits (dating to 450Ma), stratigraphically higher, and geochemically distinct from the Cu-Co(-U) deposits that characterize the Copperbelt. Kipushi is the largest of three Zn-Pb-Cu deposits in the Damaran-Lufilian fold belt—the others are Kabwe (Zambia) and Tsumeb (Namibia) [36]. Each of these three deposits has tightly-clustered “common lead” isotopic ratios, quite unlike the linear spread of highly radiogenic ratios that is typical of Cu-Co(-U) Copperbelt deposits, but Kipushi can be clearly differentiated through lead isotopes from Kabwe and Tsumeb [36]. The mineral assemblage at Kipushi shows substantial enrichment in Cu, Zn, As, Ag, Sb, and Pb in both hypogene sulfide ores and the supergene zone, which hosts a variety of copper arsenate, carbonate, oxide, phosphate, sulfate, vanadate, and chloride minerals that are often similar in color and density to malachite [32, 37, p. 134–135].

#### Magondi Belt

The metasedimentary Magondi Belt initially formed as a backarc basin from 2.2–2.0 Ga and deformed during the Magondi Orogeny between 2.0 and 1.9 Ga. This deformation generated hot brines that scavenged copper and uranium from sediments. These were redeposited to form new copper deposits [38, 39] during the same 550 Ma Pan-African orogeny that was responsible for the formation of the Copperbelt [39]. Lead isotopic data for ore samples from the Magondi Belt are radiogenic, possibly from uraninite minerals within the Dewaras group [38], and have lead isotopic data distributed around a line on the ^207^Pb/^204^Pb vs ^206^Pb/^204^Pb plot that has a much steeper slope than that of the Copperbelt (see below). More isotopic measurements of Magondi ore samples are needed to better define this trend line, and to investigate whether individual ore deposits within this mining district can be distinguished.

Copper deposits within the Magondi Belt are mostly stratiform in type, concentrated within the Deweras and Lomagundi group rocks along the eastern margins of the belt, and range from Cu only to Cu-Ag(-Au-Pd-Pt-U). There are two exceptions to this pattern, Copper Queen and Copper King, which are unique Zn-Pb-Cu-Fe-Ag deposits located in the western margin of the Magondi Belt.

### Typology and chronology of copper ingots in South-Central Africa

Archaeological interest in copper ingots in central and southern Africa began in the 1960’s with the discovery of ingots in burials at Sanga in the Upemba Depression of southern DRC [40, 41] and at Ingombe Ilede in the Zambezi Valley [22]. The earliest examples of copper ingots from central and southern Africa are small rectangular “Ia” type [42] ingots which date between the 5^th^ and 7^th^ centuries cal CE and have been found at sites in the Copperbelt and at Kumadzulo (**Fig 2**) [43, 44]. Also included in the Ia ingot type are “fishtail” style ingots which appear to be an intermediate shape between these early rectangular bars and the later *croisette* (“small cross”) ingots [42, 45, 46]. Only two fishtail ingots are known, one from Kamusongolwa Kopje and the other from Luano Main Site [45, 47], and the age of both ingots is poorly constrained between the 9^th^– 12^th^ centuries cal CE. Most ingots dating before the 12^th^ century CE are partial objects, and we can therefore reasonably infer that rectangular and fishtail ingots were traded or moved as raw material [45, p. 115–118].

After the 9^th^ century cal CE there was major expansion of the production and consumption of copper, concomitant with the introduction of the new HIH ingot type [48–50]. The production of this ingot type marks the concrete starting point for the *croisette* ingot shape, as defined in the typology published by de Maret [42]. The HIH ingot is typically 7–20 cm in length and is H-shaped, with two pairs of arms extending outward in opposite directions from an elongated central join (**Fig 2**). The chronology of HIH ingot production (9^th^-14^th^ century cal CE) was defined using examples exclusively from the Copperbelt and Upemba Depression [42], but HIH ingots are distributed from the Upemba Depression to Great Zimbabwe, mirroring the distribution of HIH ingot molds [46, 51]. Analysis of dated HIH depositional contexts in the Upemba Depression suggests that they were initially used as a raw material between the 9^th^-13^th^ centuries cal CE but were consumed as a prestige good in the 14^th^ century cal CE, as evidenced by finds of whole ingots in Kambabian-period burials [42, p. 143–144]. HIH ingots first appear in the archaeological record of Zimbabwe at this time as well, and all documented examples are whole ingots [51, p. 1008].

**Fig 3:**
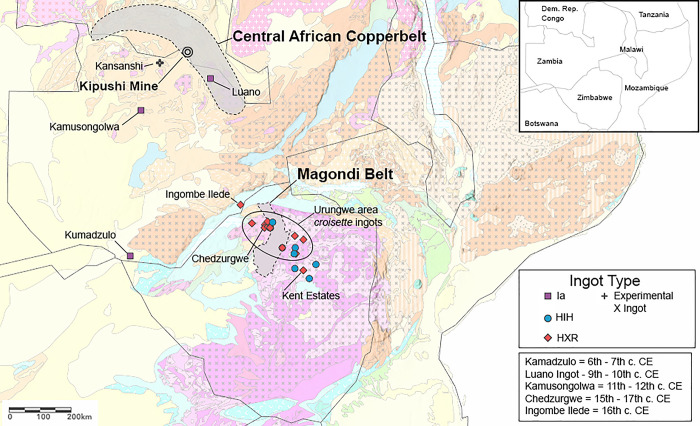
Highlighted archaeological sites, geological districts, and geological mines from the text. Geological basemap adapted from Thiéblemont et al. [20].

The 14^th^ century CE also saw the development of two separate ingot circulation spheres. Small HX and HH *croisette* ingots (0.5-7cm; **Fig 2**) and molds have been found only in the western Copperbelt and in the Upemba Depression. The much larger HXR *croisette* ingots (20–30 cm in length and 3.0–5.5 kg; **Fig 2**) are found in the eastern Copperbelt, at Ingombe Ilede in the Zambezi Valley, in northern Zimbabwe, and in a single hoard of 8 ingots in the Dedza area of Malawi [46, Fig 3]. Molds for HXR ingots are almost exclusively found in the eastern Copperbelt, including at Kipushi [2, 46], but a single HXR mold was recovered from northern Zimbabwe [51]. These HXR ingots are shaped like an X, with four arms radiating outward from a center join, a raised flange running along the outer edge of the entire ingot, and a patterned center marking (see **S2 Appendix**; [42, Fig 8, 51, Fig 1]). HXR ingots have not been reported anywhere in the DRC.

HXR ingots became a focus of archaeological attention with the discovery of the rich burials at Ingombe Ilede [22], which included eight HXR ingots, followed by the excavation of two additional HXR ingots at Chedzurgwe [52] (**Fig 3**). Based on these discoveries, Garlake set out to document finds of *croisette* and other copper ingots within northern Zimbabwe. He listed 62 examples of *croisette* ingots from 31 locations within northern Zimbabwe, the majority of which fell within the distribution zone of Ingombe Ilede type ceramics [52]; all of these were surface finds. While documenting these ingots, Garlake and J.D. White also recorded oral histories related to a group known as the Va-Mbara, who were remembered as renowned metal workers from the Urungwe area and who traded copper to the Mutapa state [52, 53]. Sixteenth century descriptions of the *Mobara* people by Portuguese explorer Antonio Fernandes are strikingly similar to the Va-Mbara and are recorded to have come from the land of “Ambar” to trade *aspas de cobre* (copper crosses) to the Mutapa state [52].

The provenance of these copper crosses, now thought to be the HXR ingots, has been debated ever since. Fernandes believed these ingots were produced near the “copper” rivers of “Manyconguo,” likely a reference to the Niari basin ore deposits near the mouth of the Congo River that were exploited by the Kongo State and its ruler ManiKongo [54], while Garlake hypothesized that these ingots were made locally at the Magondi Belt copper mines in Zimbabwe. The theory of Fernandes can be dismissed because the Niari deposits are 2100 km in a straight line from the upper Zambezi, but the hypothesis put forth by Garlake was a distinct possibility, as was the suggestion by Bisson [2] that these ingots likely source to the Copperbelt. Swan [8, 51] also argued that the HXR ingots in northern Zimbabwe were made of copper from the Magondi Belt. Until recently there was no way to decide between these hypotheses.

There are few dates for the larger HXR ingots, but on present archaeological and historical evidence they are thought to have been manufactured between the 14^th^ and 18^th^ centuries CE [42, 46]. The distribution of these ingots in northern Zimbabwe mostly matches the distribution of Ingombe Ilede style ceramics, though some have been found in areas associated with Musengezi and Mutapa culture sites [51, 52]. Like the HX and HH ingots, the HXR ingot type was clearly used as a prestige good and form of currency, as evidenced both by the 16^th^ century CE Portuguese descriptions of “copper *aspas*” trade and their deposition in burials at Ingombe Ilede [22, 46, 51, 52]. The recovery of partial HXR ingots also suggests that they were sometimes used as raw material to produce smaller copper artifacts.

The HX, HH, and HXR ingot types appear to have fallen out of favor at some point in the 18^th^ century and were replaced by the flat, un-flanged, X-shaped *handa* ingot, and by the “I” shaped Ib and Ic ingots, which weighed up to 30 kg [42, 46] (**Fig 2**). Both were frequently described by nineteenth-century European explorers in what are now Malawi, Zambia, and DRC, but they have not been recorded on the Zimbabwean plateau.

Other copper ingot types from southern Africa include the *lerale* and *musuku* ingot types from South Africa, nail head ingots, and more informal bun and bar ingots [55–57]. These other ingot types date between the 12^th^ and 20^th^ century CE and are more common in parts of Zimbabwe and South Africa. Provenance of ingots within these types will be discussed in a future publication.

### Lead isotope analysis

All copper ores contain trace amounts of lead, whose isotopic ratios are not altered by smelting and vary depending on the age, type, and other geological characteristics of the deposit. Lead isotopic analysis (LIA) has been a popular approach in Europe and the Mediterranean since the 1980’s to infer the geological sources of copper artifacts [58, 59]. A pioneering attempt to use LIA in southern Africa was made in 2005 by Suzanne Young, who used low-resolution quadrupole inductively coupled mass spectrometry (Q-ICP-MS) to measure ^207^Pb/^206^Pb ratios on ores and artifacts from Namibia [60]. The first use of high-resolution multi-collector mass spectrometry (MC-ICP-MS) for LIA in southern African archaeology was in 2008 on tin ingots from South Africa and bronze from Botswana [61]. The use of high-resolution MC-ICP-MS instrumentation for LIA is now standard because it allows for the simultaneous collection of multiple isotopes, produces data equivalent in precision to TIMS with double or triple spiking, and can correct for mass fractionation during measurement by thallium spiking [62–65].

## Samples and methods

A total of 34 samples were analyzed at the University of Arizona using ICP-MS instrumentation for lead isotopes and chemistry, 33 of which were archaeological samples collected from the Museum of Human Sciences in Harare, Zimbabwe and the Livingstone Museum in Livingstone, Zambia in 2019 (**Table 1; Fig 3**). These ingots are representative of the diversity of Copperbelt ingots in de Maret’s [42] typology that were distributed south to Zambia and Zimbabwe, and are roughly a third of the total number of these ingots documented in Zambia and Zimbabwe [42, 46, 51, 53]. All necessary permits were obtained for the described study, which complied with all relevant regulations (see **S1 Appendix**). Additional information regarding the ethical, cultural, and scientific considerations specific to inclusivity in global research is included in the **S4 Appendix**.

**Table 1:** Associated information for each sample. Further details for each sample are presented in the **S2 Appendix.**

Sample ID	Ingot Type	Country	Site	Group	Museum	Museum Number	Reference
Zim-Dul-1	HIH	Zimbabwe	Dunlorne Farm	-	ZMHS	-	-
Zim-Kent-1	HXR	Zimbabwe	Kent Estates	-	ZMHS	-	-
Zim-Riv-1	HIH	Zimbabwe	Riverside Farm	-	ZMHS	-	-
Zim-Riv-2	HIH	Zimbabwe	Riverside Farm	-	ZMHS	-	-
Zim-Ryd-1*	HXR	Zimbabwe	Chedzurgwe (Rydings)	Ingombe Ilede	ZMHS	QMIA 4462	[52]
Zim-ZMHS-1	HXR	Zimbabwe	Yeadon Farms	-	ZMHS	QMIA 4461	[51]
Zim-ZMHS-2	HXR	Zimbabwe	Chedzurgwe (Rydings)	Ingombe Ilede	ZMHS	QMIA 4462	[52]
Zim-ZMHS-4	HIH	Zimbabwe	Karoi area	-	ZMHS	QMIA 4464	[51]
Zim-ZMHS-5	HXR	Zimbabwe	Unknown	-	ZMHS	QMIA 4463	-
Zim-ZMHS-6	HXR	Zimbabwe	Zave	-	ZMHS	QMIA 4455	[51]
Zim-ZMHS-7	HXR	Zimbabwe	Kashwao East	-	ZMHS	QMIA 4457	[51]
Zim-ZMHS-8	HIH	Zimbabwe	Zave	-	ZMHS	QMIA 4456	[51]
Zim-ZMHS-9	HIH	Zimbabwe	Graniteside	Harare	ZMHS	QMIA 4459	[51]
Zim-ZMHS-10	HIH	Zimbabwe	Makwiro	-	ZMHS	QMIA 4458	[51]
Zim-ZMHS-11	HXR	Zimbabwe	Zave	-	ZMHS	QMIA 4454	[51]
Zim-ZMHS-12	HXR	Zimbabwe	Karoi Dixie farm	-	ZMHS	QMIA 3068	[51]
Zim-ZMHS-13	HXR	Zimbabwe	Chedzurgwe (Rydings)	Ingombe Ilede	ZMHS	QMIA 4451	[52]
Zim-ZMHS-14	HXR	Zimbabwe	Bassett Farm	-	ZMHS	QMIA 4453	[51]
Zim-ZMHS-15	HXR	Zimbabwe	Karoi area	-	ZMHS	QMIA 4467	[51]
Zim-ZMHS-16	HXR	Zimbabwe	Karoi area	-	ZMHS	QMIA 4465	[51]
Zim-ZMHS-18	HIH	Zimbabwe	Horizon Farm	-	ZMHS	QMIA 4498	[51]
Zim-ZMHS-19	HXR	Zimbabwe	Karoi area	-	ZMHS	QMIA 4496	[51]
Zim-ZMHS-21	HXR	Zimbabwe	Gil Gil Mine	-	ZMHS	QMIA 4478	[51]
Zim-ZMHS-22	HXR	Zimbabwe	Easter Parade Farm	-	ZMHS	-	-
Zim-ZMHS-23	HIH	Zimbabwe	Mwami	-	ZMHS	-	[51]
Zim-ZMHS-24	HIH	Zimbabwe	Beatrice area	-	ZMHS	QMIA 4484	[51]
Zim-Ship-1	HIH	Zimbabwe	Shipton Farm	-	ZMHS	-	-
Zam-II-7	HXR	Zambia	Ingombe Ilede	Ingombe Ilede	Livingstone Museum, Livingstone	-	[22]
Zam-II-14	HXR	Zambia	Ingombe Ilede	Ingombe Ilede	Livingstone Museum, Livingstone	-	[22]
Zam-II-15	HXR	Zambia	Ingombe Ilede	Ingombe Ilede	Livingstone Museum, Livingstone	-	[22]
Zam-Kuma-1	Ia (Rectangular)	Zambia	Kumadzulo	Early Iron Age	Livingstone Museum, Livingstone	-	[44]
Zam-Kamu-1	Ia (Fishtail)	Zambia	Kamusongolwa	Early Iron Age	Livingstone Museum, Livingstone	-	[47]
Zam-Kan-19	Experimental X	Zambia	Kansanshi	Modern	-	-	-
Zam-Luano-4	Ia (Fishtail)	Zambia	Luano main site	Early Iron Age	Livingstone Museum, Livingstone	-	[45]

“-”entries denote the absence of information. ZMHS = Zimbabwe Museum of Human Sciences, Harare. * Duplicate sample to Zim-ZMHS-2.

We removed the superficial corrosion layer from each of the 34 copper ingot samples using a Dremel® rotary tool, with a new carbide cut-off wheel for each sample. We then removed approximately 0.1–0.3 g of sample, using a jewelers saw with a new steel blade for each sample. These samples were weighed on a mass balance and dissolved in a solution of 8 mL 8 mol L^-1^ (or M) HNO_3_ + 0.5 mL 29 mol L^-1^ HF to minimize precipitation or volatilization of Fe, Zn, As, Ag, Sn, and Sb, typically via complexation with chloride ions [66, 67], and refluxed overnight at 140°C. All acid dilutions were prepared from double-distilled HNO_3_, HCl, and HF, and ultrapure Milli-Q water. Once cooled, a pipetted 1 mL solution of each sample was weighed to determine sample density. The total volume of each sample was then transferred to a 12 mL Falcon^TM^ tube and each Savillex vial was rinsed with 2 mL of 8 mol L^-1^ HNO_3_ to ensure total recovery of sample solution. This was then added to the Falcon tube and the total weight of each sample solution was recorded. This quantitative transfer procedure allows for the accurate calculation of total sample volume and is used to ensure the precise measurement of elemental concentrations during ICP-MS analysis. A 0.1 mL aliquot of each solution was then taken and used to prepare a series of 100x, 1000x, and 100,000x dilutions for trace element analysis at the Arizona Laboratory for Emerging Contaminants (ALEC) laboratory ICP-MS at the University of Arizona. Cr, Fe, Co, Ni, Cu, Zn, As, Se, Mo, Ag, Cd, Sn, Sb, and Pb were measured by Dr. Mary-Kay Amistadi on an Elan DRC-II ICP-MS instrument, and values are reported in μg g^-1^ (ppm). The method detection limit values for these 15 elements are also reported in **Table 2**, however these values are reported in μg L^-1^. All values below detection limits were culled prior to converting the sample data to μg g^-1^. A custom-made solution from High Purity Standard and Claritas PPT^®^ Grade ICP-MS Instrument Calibration Standard 2 from Spex CirtiPrep were run with every batch for quality control. A full discussion of our choices for multivariate statistical analysis of chemical data can be found in the **S1 Appendix**.

**Table 2:** Chemical data for samples. All values are reported in μg g^-1^. Detection limits are included in the second row, however these values are reported in μg L^-1^ of dissolved sample in solution. Thus, all values below detection limits were culled prior to converting the sample data to μg kg^-1^ (ppb), μg g^-1^ (ppm), and wt% in the solid sample. <D.L = less than detection limits.

Sample ID	Ingot Type	Site	Provenance	Cr	Fe	Co	Ni	Zn	As	Se	Mo	Ag	Cd	Sn	Sb	Pb
detection limit in μg L^-1^				0.043	0.011	0.619	0.006	0.032	0.014	0.025	0.542	0.010	0.003	0.002	0.019	0.005
Zim-Dul-1	HIH	Dunlorne Farm	Copperbelt	<D.L	5	54	7	2	17	<D.L	<D.L	<1	<1	1	<1	<1
Zim-Kent-1	HXR	Kent Estates	Magondi Belt	<D.L	490	1	30	4	8	145	<1	1572	<D.L	1	<1	15
Zim-Riv-1	HIH	Riverside Farm	Copperbelt	<D.L	10	140	20	2	58	<D.L	<D.L	25	<D.L	2	1	1
Zim-Riv-2	HIH	Riverside Farm	Copperbelt	<D.L	<D.L	5	10	2	16	<D.L	<D.L	16	<D.L	1	<1	<1
Zim-Ryd-1	HXR	Chedzurgwe (Rydings)	Kipushi	<D.L	11	<1	2	30	304	4	<D.L	1532	<1	<1	12	48
Zim-ZMHS-1	HXR	Yeadon Farms	Kipushi	<D.L	53	<1	1	39	240	<D.L	<D.L	1885	<1	1	2	14
Zim-ZMHS-2	HXR	Chedzurgwe (Rydings)	Kipushi	<D.L	12	<1	2	37	608	6	<D.L	1093	1	<1	39	293
Zim-ZMHS-4	HIH	Karoi area	Kipushi	<D.L	51	<1	2	146	1887	<D.L	<D.L	617	<1	<1	23	1465
Zim-ZMHS-5	HXR	Unknown	Copperbelt	<1	158	97	8	4	44	4	<D.L	91	<1	1	1	4
Zim-ZMHS-6	HXR	Zave	Kipushi	<D.L	60	<1	2	109	624	3	<D.L	1349	<1	<1	9	91
Zim-ZMHS-7	HXR	Kashwao East	Kipushi	<D.L	15	<D.L	1	56	1694	4	<D.L	1445	1	<1	11	149
Zim-ZMHS-8	HIH	Zave	Copperbelt	<D.L	28	50	8	2	53	<D.L	<1	96	<D.L	<1	<1	<1
Zim-ZMHS-9	HIH	Graniteside	Copperbelt	<D.L	38	38	42	4	72	<D.L	<D.L	177	<1	1	<1	1
Zim-ZMHS-10	HIH	Makwiro	Kipushi	<D.L	27	<1	6	28	1443	22	<D.L	564	<D.L	1	59	1372
Zim-ZMHS-11	HXR	Zave	Kipushi	<D.L	17	<1	2	13	2515	11	<D.L	1759	<1	1	111	354
Zim-ZMHS-12	HXR	Karoi Dixie farm	Kipushi	<D.L	9	<1	2	95	917	11	<D.L	1617	1	<1	27	1128
Zim-ZMHS-13	HXR	Chedzurgwe (Rydings)	Kipushi	<D.L	17	<D.L	1	31	324	4	<D.L	1608	<1	<1	14	51
Zim-ZMHS-14	HXR	Bassett Farm	Kipushi	<D.L	11	<D.L	4	29	338	6	<D.L	843	<1	<1	2	126
Zim-ZMHS-15	HXR	Karoi area	Kipushi	<D.L	47	<1	3	131	740	<D.L	<D.L	1257	<1	<1	29	176
Zim-ZMHS-16	HXR	Karoi area	Copperbelt	<D.L	13	144	8	2	58	<D.L	<1	11	<D.L	<1	<1	<1
Zim-ZMHS-18	HIH	Horizon Farm	Copperbelt	<D.L	16	2	22	3	11	<D.L	<D.L	8	<D.L	<1	<1	<1
Zim-ZMHS-19	HXR	Karoi area	Kipushi	<D.L	29	<D.L	1	33	585	9	<1	1076	<1	<1	10	61
Zim-ZMHS-21	HXR	Gil Gil Mine	Kipushi	<D.L	17	<1	3	33	477	9	<D.L	1654	<1	<1	18	187
Zim-ZMHS-22	HXR	Easter Parade Farm	Copperbelt	<D.L	28	112	11	4	28	<D.L	<D.L	7	<1	<D.L	<1	1
Zim-ZMHS-23	HIH	Mwami	Kipushi	<D.L	19	<1	6	60	1000	10	<D.L	967	<D.L	<1	62	709
Zim-ZMHS-24	HIH	Beatrice area	Copperbelt	<D.L	32	16	32	5	10	<D.L	<D.L	5	<D.L	<D.L	<1	1
Zim-Ship-1	HIH	Shipton Farm	Copperbelt	<D.L	14	74	15	2	50	<D.L	<D.L	1	<D.L	1	1	<1
Zam-II-7	HXR	Ingombe Ilede	Kipushi	<D.L	53	<1	2	73	591	7	<D.L	88	<1	37	24	142
Zam-II-14	HXR	Ingombe Ilede	Copperbelt	<D.L	12	127	5	2	28	<D.L	<D.L	10	<1	19	<1	<1
Zam-II-15	HXR	Ingombe Ilede	Kipushi	<D.L	13	<1	3	63	483	3	<D.L	1966	<1	3	26	54
Zam-Kuma-1	Ia (Rectangular)	Kumadzulo	Copperbelt	<1	71	204	11	2	43	4	<1	<1	<1	1	<1	2
Zam-Kamu-1	Ia (Fishtail)	Kamusongolwa	Copperbelt	1	32	17	11	9	13	<D.L	<1	<1	<1	1	<1	<1
Zam-Kan-19	Experimental X	Kansanshi	Kansanshi	1	85	<1	31	3	1	4	<D.L	<1	<1	1	<1	<1
Zam-Luano-4	Ia (Fishtail)	Luano main site	Copperbelt	<1	27	5	11	6	13	<D.L	<D.L	<1	<1	<D.L	<D.L	<1

The remaining sample was then re-transferred to its Savillex vial and evaporated to dryness at 150°C. Once evaporated and cooled, samples were re-dissolved in 2 mL of 8 mol L^-1^ HNO_3_ and allowed to reflux overnight at 120°C. This solution was then separated using Bio-Rad disposable anion exchange columns loaded with Eichrom Sr-spec resin and eluted with various concentrations of twice distilled HCl and HNO_3_ to isolate the lead portion of each solution [68, 69]. The resulting solution was then evaporated to dryness and 1 mL of 2% HNO_3_ was added to each sample vial refluxed on a hotplate at 120°C overnight. Samples were then analyzed on the GV Instruments IsoProbe multi-collector inductively coupled plasma mass spectrometer (MC-ICP-MS) housed in the Department of Geosciences and the University of Arizona (**Table 3**). Data was corrected based on published values for the standard NBS981 [70] and all samples were empirically normalized with a thallium (Tl) spike using the exponential law correction [63]. A mercury (^204^Hg) correction is also typically applied to correct for interference on the ^204^Pb signal, however Hg contents of the carrier gas were typically very low. Only the HIH sample Zim-ZMHS-18 required a mercury correction in this study. Procedural blanks were also measured, and all contained <250 pg of lead.

**Table 3:** LIA values for samples.

Sample ID	Ingot Type	Site	Lead (μg g^-1^)	^208^Pb/^206^Pb	2σ	^207^Pb/^206^Pb	2σ	^206^Pb/^204^Pb	2σ	^207^Pb/^204^Pb	2σ	^208^Pb/^204^Pb	2σ	Provenance
Zim-Dul-1	HIH	Dunlorne Farm	<1	1.138	0.0003	0.485	0.0000	34.279	0.0040	16.623	0.0035	39.026	0.0116	Copperbelt
Zim-Kent-1	HXR	Kent Estates	15	1.534	0.0003	0.687	0.0000	24.393	0.0040	16.754	0.0035	37.428	0.0116	Magondi Belt
Zim-Riv-1	HIH	Riverside Farm	1	2.023	0.0002	0.829	0.0000	18.955	0.0014	15.717	0.0014	38.339	0.0038	Copperbelt
Zim-Riv-2	HIH	Riverside Farm	<1	1.935	0.0002	0.773	0.0000	20.448	0.0041	15.817	0.0040	39.567	0.0101	Copperbelt
Zim-Ryd-1	HXR	Chedzurgwe (Rydings)	48	2.084	0.0002	0.866	0.0000	18.060	0.0014	15.634	0.0014	37.639	0.0038	Kipushi
Zim-ZMHS-1	HXR	Yeadon Farms	14	2.085	0.0002	0.865	0.0000	18.067	0.0014	15.634	0.0014	37.663	0.0038	Kipushi
Zim-ZMHS-2	HXR	Chedzurgwe (Rydings)	293	2.084	0.0002	0.866	0.0000	18.058	0.0014	15.630	0.0014	37.630	0.0038	Kipushi
Zim-ZMHS-4	HIH	Karoi area	1465	2.085	0.0002	0.866	0.0001	18.052	0.0019	15.629	0.0009	37.628	0.0045	Kipushi
Zim-ZMHS-5	HXR	Unknown	4	2.051	0.0002	0.843	0.0001	18.604	0.0019	15.684	0.0009	38.149	0.0045	Copperbelt
Zim-ZMHS-6	HXR	Zave	91	2.085	0.0002	0.866	0.0001	18.047	0.0019	15.626	0.0009	37.624	0.0045	Kipushi
Zim-ZMHS-7	HXR	Kashwao East	149	2.084	0.0002	0.866	0.0001	18.048	0.0019	15.626	0.0009	37.617	0.0045	Kipushi
Zim-ZMHS-8	HIH	Zave	<1	1.772	0.0001	0.726	0.0000	21.868	0.0022	15.881	0.0020	38.755	0.0053	Copperbelt
Zim-ZMHS-9	HIH	Graniteside	1	1.999	0.0001	0.821	0.0000	19.149	0.0022	15.715	0.0020	38.283	0.0053	Copperbelt
Zim-ZMHS-10	HIH	Makwiro	1372	2.086	0.0001	0.866	0.0000	18.045	0.0022	15.634	0.0020	37.636	0.0053	Kipushi
Zim-ZMHS-11	HXR	Zave	354	2.085	0.0001	0.866	0.0000	18.051	0.0022	15.633	0.0020	37.641	0.0053	Kipushi
Zim-ZMHS-12	HXR	Karoi Dixie farm	1128	2.086	0.0001	0.866	0.0000	18.047	0.0022	15.634	0.0020	37.640	0.0053	Kipushi
Zim-ZMHS-13	HXR	Chedzurgwe (Rydings)	51	2.084	0.0001	0.866	0.0001	18.057	0.0037	15.630	0.0028	37.632	0.0083	Kipushi
Zim-ZMHS-14	HXR	Bassett Farm	126	2.086	0.0001	0.866	0.0001	18.039	0.0037	15.628	0.0028	37.631	0.0083	Kipushi
Zim-ZMHS-15	HXR	Karoi area	176	2.084	0.0001	0.866	0.0001	18.061	0.0037	15.634	0.0028	37.640	0.0083	Kipushi
Zim-ZMHS-16	HXR	Karoi area	<1	1.968	0.0001	0.798	0.0001	19.780	0.0037	15.783	0.0028	38.929	0.0083	Copperbelt
Zim-ZMHS-18	HIH	Horizon Farm	<1	1.927	0.0002	0.772	0.0000	20.511	0.0041	15.825	0.0040	39.523	0.0101	Copperbelt
Zim-ZMHS-19	HXR	Karoi area	61	2.086	0.0001	0.866	0.0000	18.045	0.0023	15.632	0.0024	37.634	0.0068	Kipushi
Zim-ZMHS-21	HXR	Gil Gil Mine	187	2.085	0.0001	0.866	0.0000	18.053	0.0023	15.633	0.0024	37.641	0.0068	Kipushi
Zim-ZMHS-22	HXR	Easter Parade Farm	1	2.016	0.0002	0.812	0.0000	19.394	0.0041	15.755	0.0040	39.091	0.0101	Copperbelt
Zim-ZMHS-23	HIH	Mwami	709	2.085	0.0001	0.866	0.0000	18.049	0.0023	15.632	0.0024	37.639	0.0068	Kipushi
Zim-ZMHS-24	HIH	Beatrice area	1	2.014	0.0001	0.807	0.0000	19.517	0.0023	15.756	0.0024	39.303	0.0068	Copperbelt
Zim-Ship-1	HIH	Shipton Farm	<1	1.971	0.0001	0.770	0.0001	20.607	0.0019	15.862	0.0016	40.607	0.0030	Copperbelt
Zam-II-7	HXR	Ingombe Ilede	142	2.085	0.0004	0.866	0.0001	18.057	0.0022	15.635	0.0028	37.647	0.0073	Kipushi
Zam-II-14	HXR	Ingombe Ilede	<1	1.888	0.0002	0.761	0.0000	20.809	0.0031	15.836	0.0028	39.287	0.0097	Copperbelt
Zam-II-15	HXR	Ingombe Ilede	54	2.084	0.0002	0.866	0.0000	18.055	0.0031	15.631	0.0028	37.633	0.0097	Kipushi
Zam-Kuma-1	Ia (Rectangular)	Kumadzulo	2	1.940	0.0001	0.792	0.0001	19.937	0.0030	15.799	0.0027	38.667	0.0062	Copperbelt
Zam-Kamu-1	Ia (Fishtail)	Kamusongolwa	<1	1.576	0.0002	0.648	0.0000	24.841	0.0041	16.088	0.0040	39.137	0.0101	Copperbelt
Zam-Kan-19	Experimental X	Kansanshi	<1	1.980	0.0002	0.811	0.0001	19.359	0.0027	15.699	0.0029	38.338	0.0071	Kansanshi
Zam-Luano-4	Ia (Fishtail)	Luano main site	<1	1.984	0.0003	0.796	0.0001	19.812	0.0066	15.781	0.0043	39.316	0.0102	Copperbelt

## Results

### Lead isotopes

#### Rectangular and fishtail ingots (type “Ia”)

**Fig 4:**
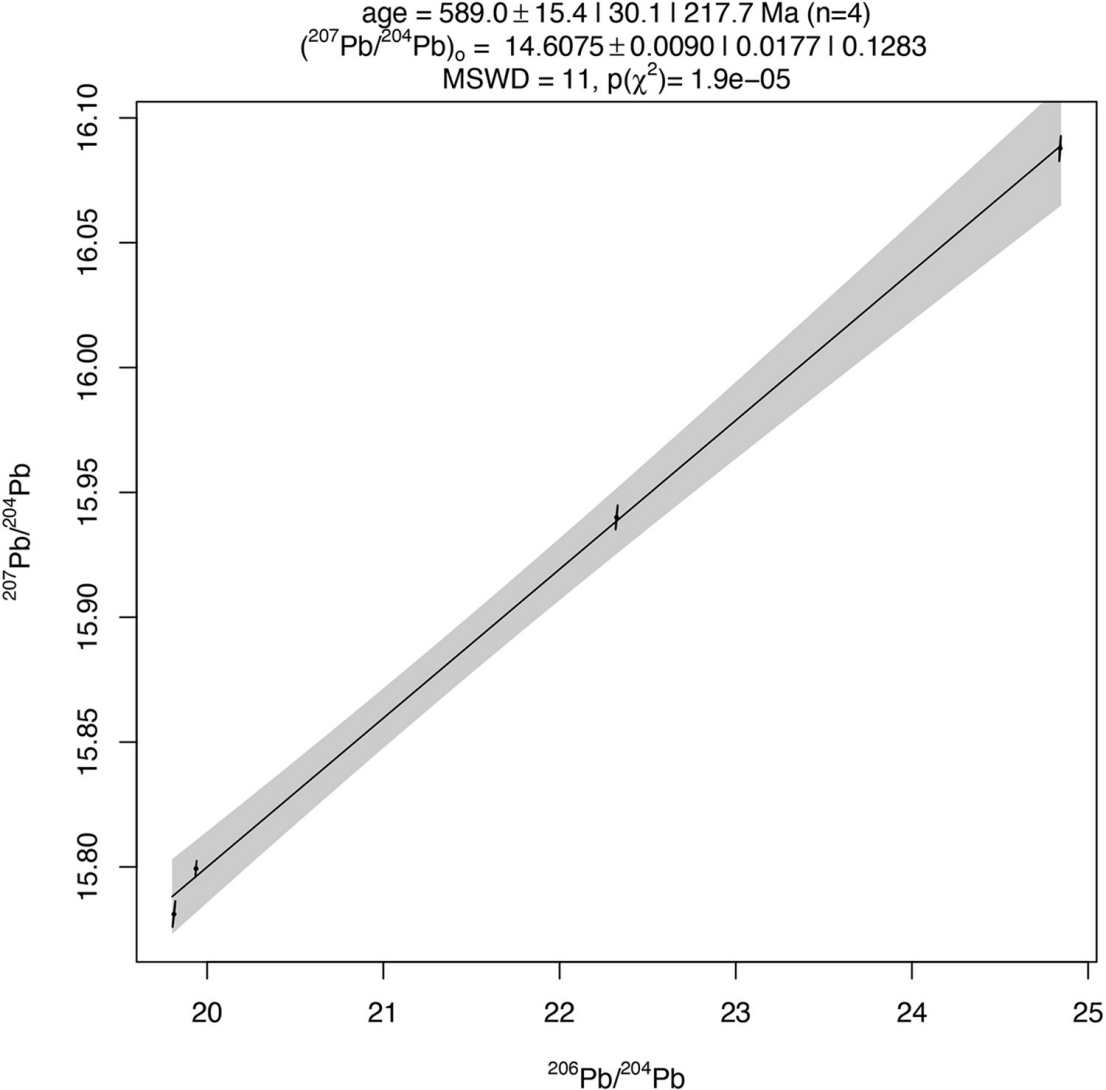
“Ia” type ingot isochron. Produced from fishtail ingots from Kamusongolwa and Luano, the rectangular ingot from Kumadzulo, and the Luano ingot sample from Rademakers et al. [24]. 0.0177 is the 100(1- α)% confidence interval for the ^207^Pb/^204^Pb intercept. 0.1283 is the studentised 100(1-α)% confidence interval for u with overdispersion. The gray band around the regression line represents the confidence interval. MSWD is Mean Square of the Weighted Deviates, and gives an indication of the mean distance of points from the line. Isochron calculated using the IsoplotR “three Ratio” option for Pb-Pb isochrons [71].

**Fig 5:**
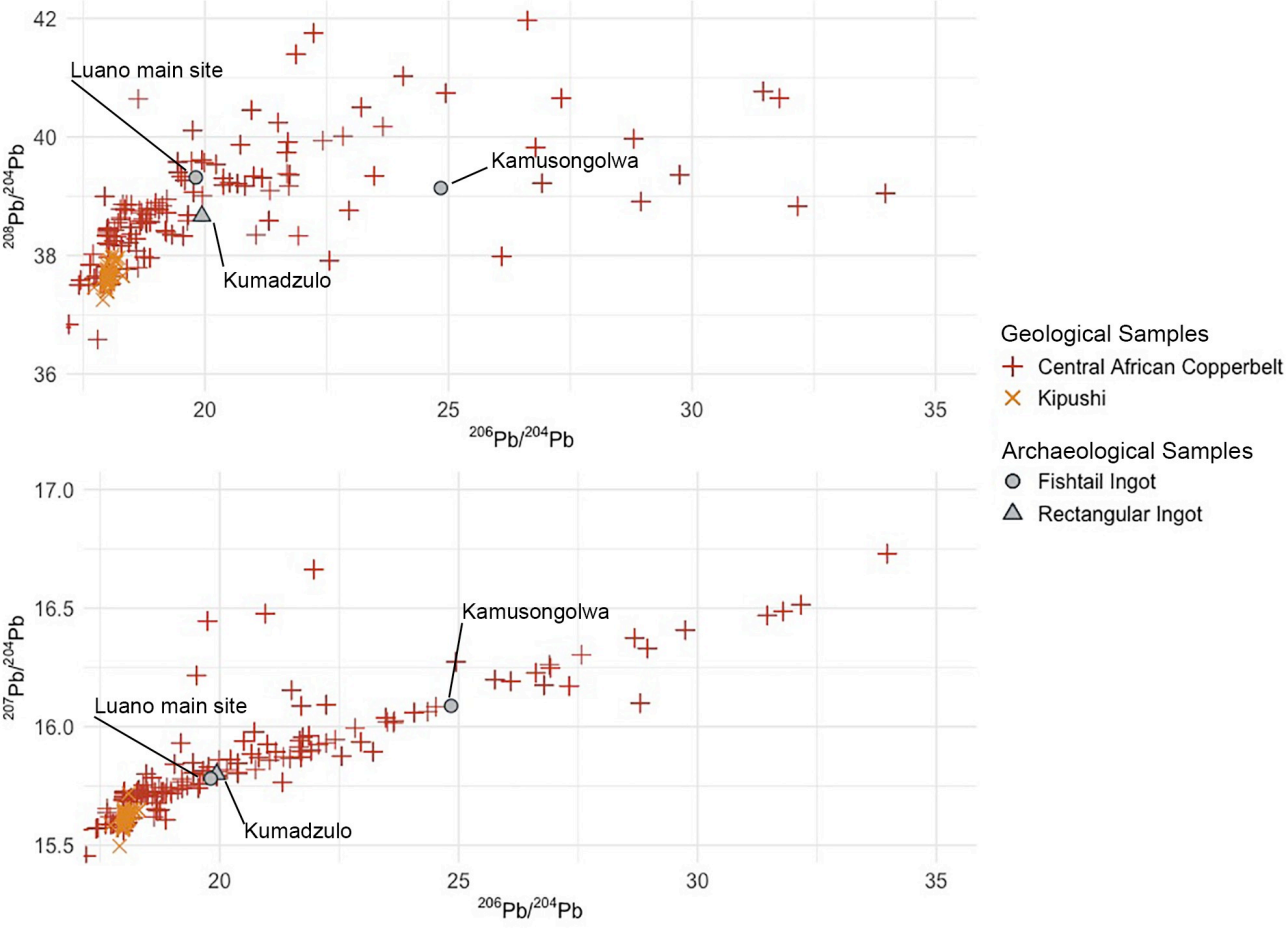
LIA data from rectangular and fishtail ingots. Ingot data is compared to geological ore data from the Central African Copperbelt. The geological data is comprised of ore samples from the Domes Region, Kafue Syncline, Katanga Core, Katanga Copperbelt, Kundelungu Plateau, and Zambian Copperbelt–all of which are genetically related. Ore data in the **S3 Appendix** from compilation of Killick et al. [59] and data produced by Stephens in 2020.

Lead isotopic data for the three rectangular and fishtail ingots (**Table 3**) range in ^206^Pb/^204^Pb from 19.81 to 24.84, in ^207^Pb/^204^Pb from 15.78 to 16.09, and in ^208^Pb/^204^Pb from 38.31 to 39.14. The rectangular ingot from Kumadzulo and the fishtail ingot from Luano have nearly identical lead isotopic data, with only slight differences in the ^208^Pb/^204^Pb ratio. The Kamusongolwa fishtail ingot has a similar ^208^Pb/^204^Pb ratio to the Luano fishtail ingot but has much higher ^206^Pb/^204^Pb and ^207^Pb/^204^Pb values. These ingots form a linear array on the ^206^Pb/^204^Pb vs ^207^Pb/^204^Pb plot, and isochron analysis produces an age of 584.5 ± 15.9 Ma, with a mean square of weighted deviates (MSWD) of 21. Rademakers et al. [24] also analyzed one rectangular ingot, and the isochron age is only slightly changed to 589 ± 15.4 Ma with an improved MSWD of 11 if this sample is included (**Fig 4**). The pattern of radiogenic lead isotopic data on the ^206^Pb/^204^Pb and ^207^Pb/^204^Pb ratios, a ^208^Pb/^204^Pb ratio ranging from 37–42, and an isochron age in the range of 650–550 Ma is typical of many ore deposits in the Copperbelt and was observed by both Rademakers et al. [24] and Stephens et al. [33]. There is broad isotopic agreement between these three samples and Copperbelt ores in our LIA database [59] (expanded subsequently by Stephens in the **S3 Appendix**) (**Fig 5**), but it is currently impossible to assign these samples to particular mines within the Copperbelt.

#### Croisette (HIH, HXR, and experimental “X”) ingots

**Fig 6:**
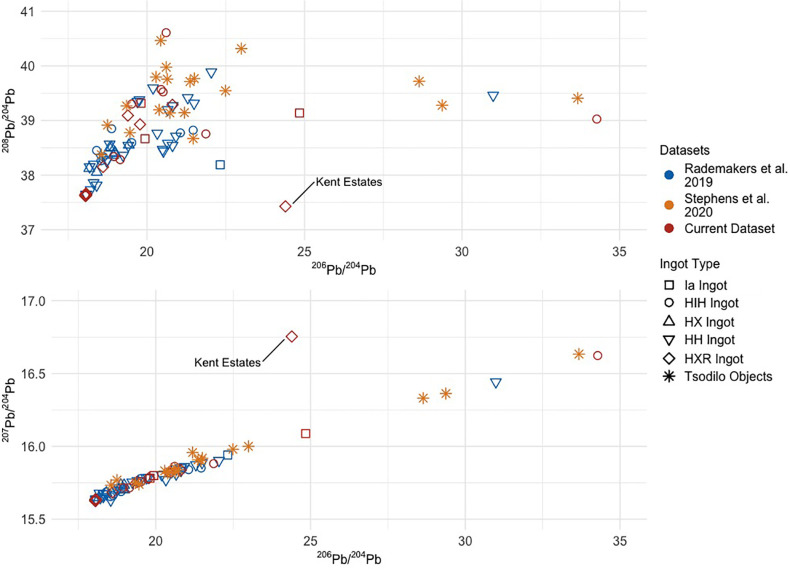
Comparison to existing archaeological data. Ia (rectangular and fishtail) and *croisette* ingot data from this study compared to ingot data from Rademakers et al. [24] on Ia (rectangular) and *croisette* ingots from the Upemba Depression. Results from these two projects agree well with one another and clearly illustrate that the Kent Estates HXR ingot was produced from a different geological source.

**Fig 7:**
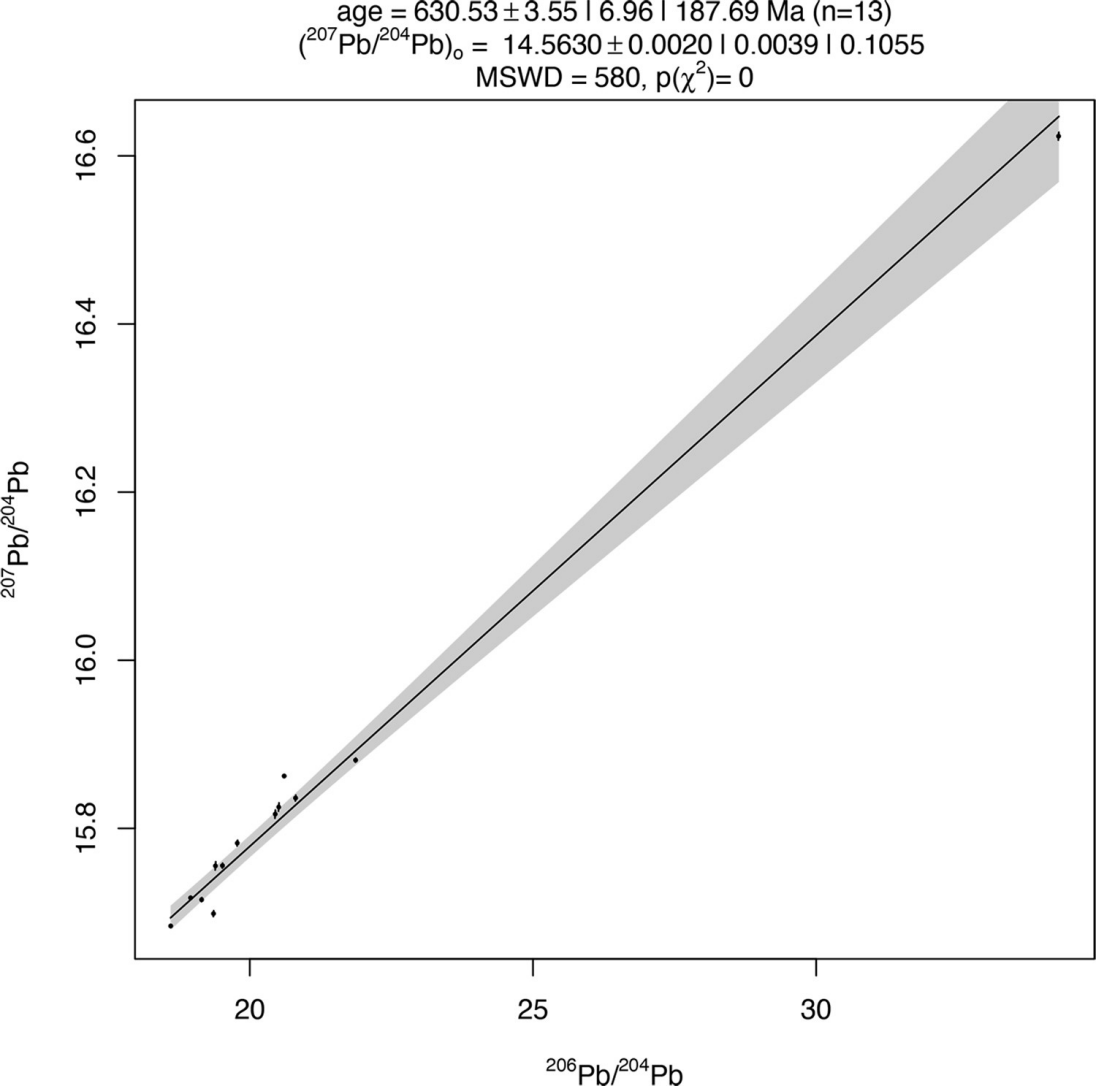
HIH and HXR ingot isochron. Produced from ingots with radiogenic lead isotopic data (^206^Pb/^204^Pb > 18.700. ^207^Pb/^204^Pb > 15.628). The Kent Estates ingot and ingots matching the Kipushi deposit were excluded from this calculation. 0.0039 is the 100(1- α)% confidence interval for the ^207^Pb/^204^Pb intercept. 0.1055 is the studentised 100(1-α)% confidence interval for u with overdispersion. The gray band around the regression line represents the confidence interval. MSWD is the Mean Square of the Weighted Deviates, and gives an indication of the mean distance of points from the line. Isochron calculated using the IsoplotR “three Ratio” option for Pb-Pb isochrons [71].

**Fig 8:**
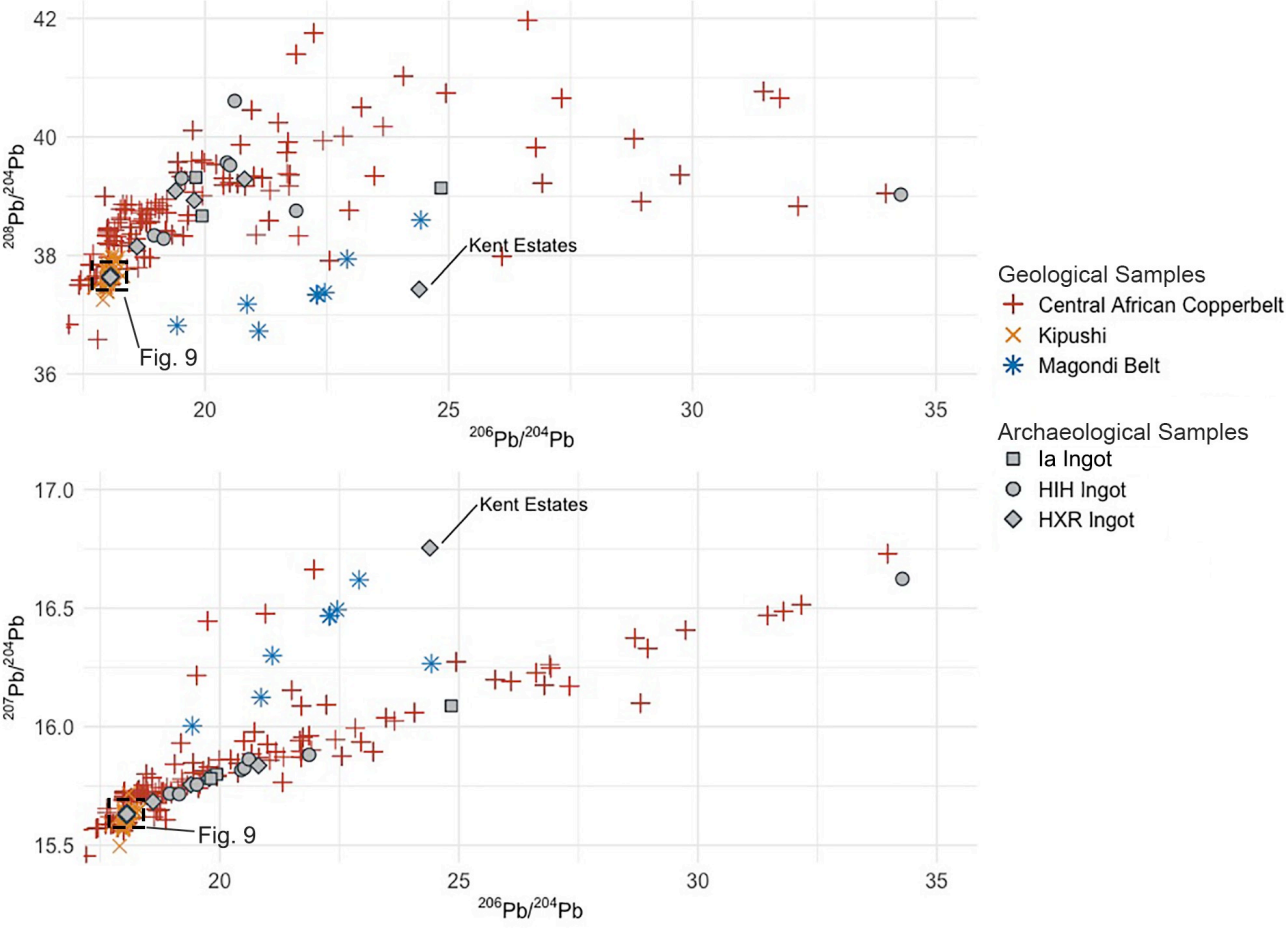
LIA data from *croisette* ingots. Comparison of HIH and HXR ingot LIA data to geological ore LIA data from the Central African Copperbelt and Magondi belt. Ore data in the **S3 Appendix** from compilation of Killick et al. [59] and data produced by Stephens in 2020. The Kent Estates HXR ingot clearly diverges from the dominant trend in Copperbelt LIA values and is a better match for the Magondi belt. However, more isotopic measurements of Magondi belt ore samples are needed to better define this trend line, and to investigate whether individual ore deposits within this mining district can be distinguished.

Lead isotopic data for the 29 *croisette* ingots from Ingombe Ilede and northern Zimbabwe and the experimental “X” ingot range in ^206^Pb/^204^Pb from 18.04 to 34.28, in ^207^Pb/^204^Pb from 15.63 to 16.76, and in ^208^Pb/^204^Pb from 37.43 to 40.61 (**Table 3**). The *croisette* ingots form two distinct groups within this range. The first group—composed of eight HIH ingots, four HXR ingots, and the experimental “X” ingot—matches previously produced data by Rademakers et al. [24], Stephens et al. [33], and the three rectangular and fishtail ingots from Zambia (**Fig 6**). These 13 samples form a linear distribution on the ^206^Pb/^204^Pb vs ^207^Pb/^204^Pb plot and have ^208^Pb/^204^Pb values between 38.08–40.61. The regression line fitted to these points gives a calculated isochron age of 627.25 ± 3.57 Ma, with an MSWD of 580 (**Fig 7**). This is within the range of ages inferred for ore formation in the Copperbelt, as noted above. There is also good agreement between isotopic values for this group and the values for Copperbelt ores (**Fig 8**). As noted above, it is currently impossible to determine a more specific provenance for these samples.

**Fig 9:**
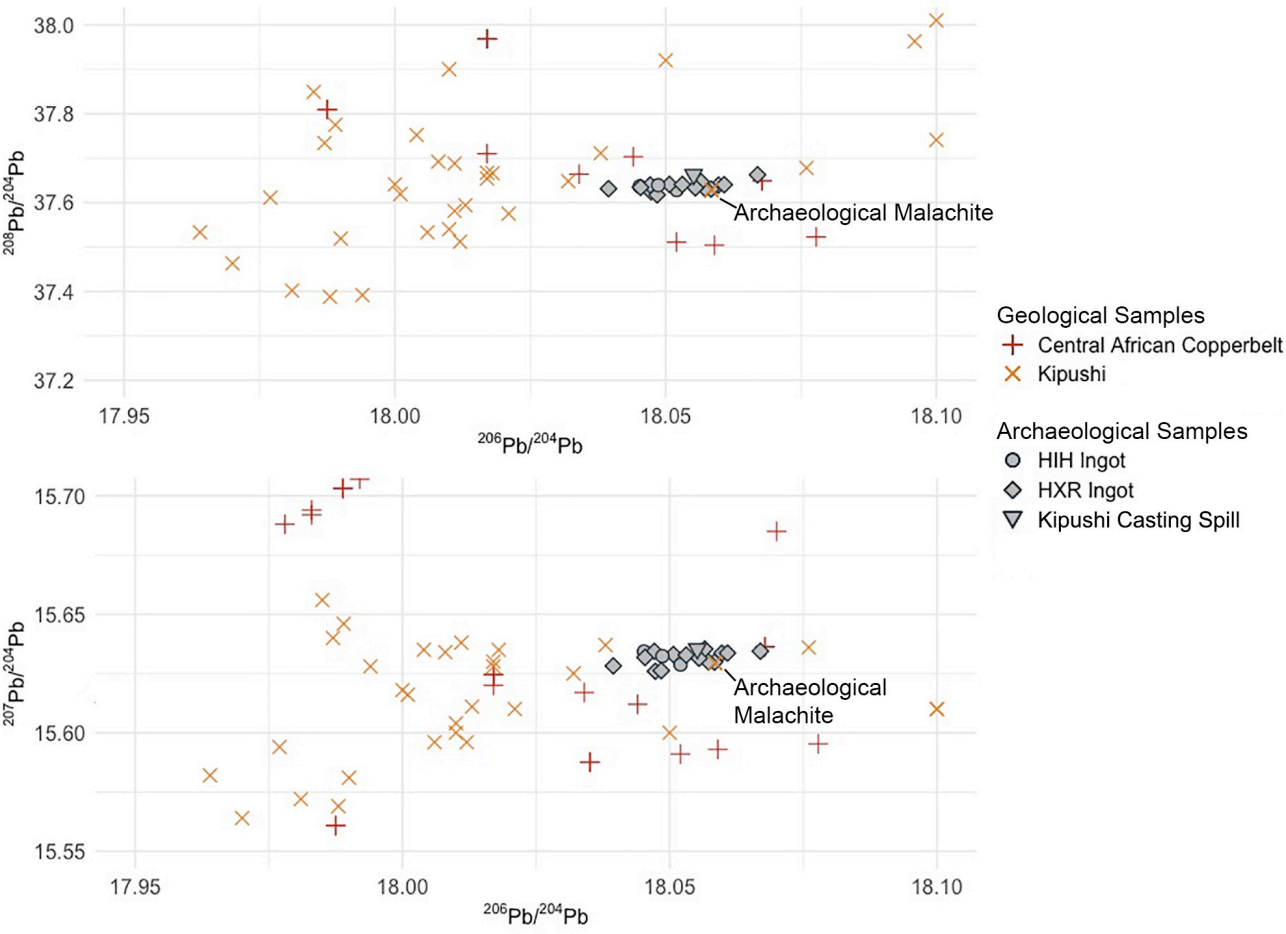
Highlighted samples from Fig 8. A zoomed-in perspective of the highlighted box from **Fig 8** shows the highly clustered group of HIH and HXR ingot samples which match to LIA data from the Zn-Pb-Cu Kipushi deposit. Note that the scale is drastically different from **Fig 8**. Included in this plot are one copper casting spill and one fragment of malachite recovered from smelting sites near Kipushi. Present in this narrow window are 30 datapoints from ore samples from the Kipushi deposit, and 9 datapoints from 7 other deposits within the Copperbelt that are closest to the Kipushi cluster. Ore data in the **S3 Appendix** from compilation of Killick et al. [59] and data produced by Stephens in 2020.

The second group in this assemblage of *croisette* ingots is composed of three HIH and 13 HXR ingots whose lead isotope data forms a tight cluster of non-radiogenic values centered around 18.05 in ^206^Pb/^204^Pb, 15.64 in ^207^Pb/^204^Pb, and 37.64 in ^208^Pb/^204^Pb. Three large HH ingots (Fe-29, K-1, K-7) analyzed by Rademakers et al. [24] from the Upemba Depression are also members of this cluster. These samples all match geological ore samples from the Kipushi deposit in the Copperbelt, which cluster around the mean values of 18.03 in ^206^Pb/^204^Pb, 15.61 in ^207^Pb/^204^Pb, and 37.67 in ^208^Pb/^204^Pb (**Fig 9**).

One HXR ingot (from Kent estates, Zimbabwe) is an outlier and does not fit with either Copperbelt or Kipushi ores. The lead isotopic data for this sample is radiogenic but has a much higher ^207^Pb/^204^Pb ratio and lower ^208^Pb/^204^Pb ratio than Copperbelt ores (**Figs 6 and 8**). The lead isotopic data for this sample matches best with the distribution of ore data from the Magondi Belt, though we acknowledge that more isotopic data from ores in the Magondi Belt are needed.

### Chemistry

The concentrations of 13 elements in each of the 34 ingots are reported in **Table 2**. We applied hierarchical cluster analysis to assess patterns in chemical similarity for our entire southern African copper ingot database (n = 46), which also includes bar, bun, *lerale*, *musuku*, and nail head ingot samples from South Africa and Zimbabwe. Based on results from the *fviz_nbclust* function in the factoextra R package, we can divide the samples into six compositional groupings. These are: 1) “Copperbelt group 1”, 2) “Phalaborwa and Magondi Belt”, 3) “Copperbelt group 2”, 4) “Kipushi”, 5) “Phalaborwa and Copper Queen”, and 6) “Phalaborwa and other”. Compositional groups were then inspected by PCA to assess variabilities within the hierarchical cluster analysis relating to source attribution, technology, and deposit geochemistry. For more detail on hierarchical cluster analysis and PCA methods, results, and cluster assignments, see discussion in the **S1 Appendix**.

#### Rectangular and fishtail ingots (type “Ia”)

**Fig 10:**
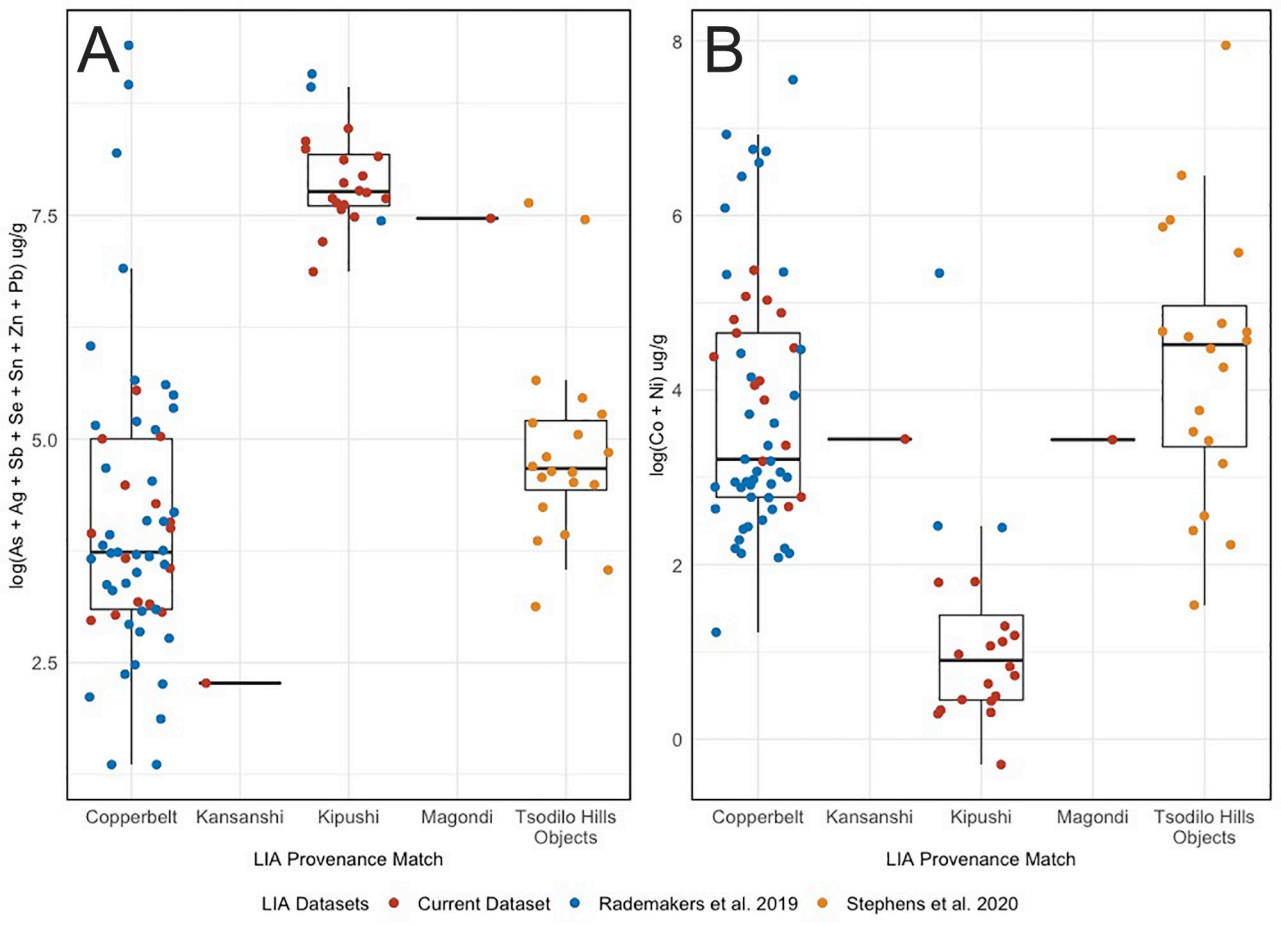
Chemistry Boxplots. Logged concentrations of chalcophile (A) and siderophile (B) elements from samples in this paper, Rademakers et al. [24], and Stephens et al. [33]. Ingots are grouped by their determined isotopic provenance, and the experimental “X” ingot is represented by the “Kansanshi” category since we know its specific provenance.

The three rectangular and fishtail ingots are extraordinarily pure (**Table 2**), with concentrations of Cr, Se, Mo, Ag, Cd, Sn, Sb, and Pb all less than 5 μg g^-1^, and concentrations of Ni, Zn, and As not exceeding 50 μg g^-1^. Cobalt and iron are by far the most concentrated elements in these samples, yet still only range between 5–204 μg g^-1^ for Co, and 27–71 μg g^-1^ for Fe. This elemental patterning, and most importantly the extremely low concentrations of Pb, was also observed in most archaeological copper samples analyzed by Rademakers et al. [24] from DRC and by Stephens et al. [33] from northern Botswana (**Fig 10**). As noted above (Fig 4) all three of these samples have radiogenic lead isotope ratios that match ores from the Copperbelt. Unfortunately, there are more than 150 Cu-Co(-U) deposits in the Copperbelt, and extensive overlaps in lead isotope ratios and trace element concentrations make it impossible at present to assign archaeological samples to individual mines. The hierarchical cluster analysis assigns these three ingots to the “Copperbelt group 1” or “Copperbelt group 2” clusters because they are depleted in chalcophile elements (see **Figs A and B in the S1 Appendix**).

#### *Croisette* (HIH, HXR, and experimental “X”) ingots

Our lead isotopic results for sampled HIH and HXR ingots, and the experimental “X” ingot split mostly into two distinct groups, whose chemical characteristics (**Table 2**) support the lead isotopic designations discussed above.

1. The first group is comprised of 13 ingots which almost all have Cr, Zn, Se, Mo, Cd, Sn, Sb and Pb concentrations under 5 μg g^-1^, Ag concentrations under 20 μg g^-1^, Ni concentrations between 5–42 μg g^-1^, and Fe values under 50 μg g^-1^. Co values, conversely, range between 2–144 μg g^-1^, with a mean of 72 μg g^-1^. All 13 of these ingots match Copperbelt ores on lead isotope ratios (Table 3, Fig 8) and their chemical compositions match those of other archaeological samples who also match Copperbelt ores on lead isotope ratios (Fig 10). These include the three rectangular and fishtail ingots, as well as the majority of samples from Rademakers et al. [24] and Stephens et al. [33]. Stephens et al. [33] also linked Co:Ni trends to different generations of Cu-Co mineralization in the Copperbelt (based on Cailteaux et al. [31]). Ingots in this first group have similar variation in their Co:Ni ratio and suggest use of ore from both first (in the Menda and Luishia facies) and second generation Cu-Co deposits. Three samples also have Ag values between 90–177 μg g^-1^, higher than the 8 μg g^-1^ average for the other 10 samples in this category. Silver has been recorded at sub-economic concentrations in the supergene zone of several Cu-Co(-U) deposits [31]. Variations in Ag concentration and Co:Ni ratio therefore hints at the exploitation of several different Cu-Co(-U) deposits to produce these ingots. While the chemistry results support our lead isotopic provenance assignments, they do little to further isolate exactly where within the Copperbelt these samples originate. The experimental “X” ingot is chemically included in this group, even though we know it was smelted using copper ore mined from the Iron Oxide Copper Gold (IOCG) Kansanshi deposit. This further illustrates the difficulty of discriminating between ore deposits in northern Zambia and the DRC, except for Kipushi (see below). The hierarchical cluster analysis assigns all ingots within this group to the “Copperbelt group 1” and “Copperbelt group 2” clusters because they are depleted in chalcophile elements (see **Figs A and B in the S1 Appendix**), once again reaffirming our lead isotopic and descriptive chemistry conclusions. We do not yet have a compelling geological explanation for the separation of “Copperbelt group 1” and “Copperbelt group 2”.
2. A second group of 16 ingots all contain much higher concentrations of Zn (13–146 μg g^-1^, mean of 61 μg g^-1^), As (240–2515 μg g^-1^, mean of 869 μg g^-1^), Ag (88–1966 μg g^-1^, mean of 1254 μg g^-1^), Sb (2–111 μg g^-1^, mean of 29 μg g^-1^), and Pb (14–1465 μg g^-1^, mean of 378 μg g^-1^), and most have concentrations of Co, Ni, Se, Mo, Cd, and Sn concentrations below 5 μg g^-1^. These 16 ingots all have lead isotope ratios that match those of ore samples from the Kipushi deposit, as do the three large HH ingot samples from Rademakers et al. [24] that have very similar chemistry to this group of ingots **(Fig 10)**. The enriched chalcophile elements (Zn, As, Ag, Sb, and Pb) in these samples could have either substituted for the Cu^2+^ ion in malachite or been introduced to the smelt through the accidental addition of other supergene copper minerals which are similar in color and density to malachite [32, 37, p. 134–135]. In our multivariate statistical analysis, this group of 16 Kipushi ingots forms an extremely tight cluster (the “Kipushi” cluster), that separates clearly from the two Copperbelt clusters (see **Figs A and B in the S1 Appendix**).

The HXR ingot from Kent Estates is the lone outlier and presents a distinctly different chemical composition than the other two *croisette* groups (**Fig 10**). This sample contains Cr, Co, Zn, As, Mo, Cd, Sn, Sb, and Pb at a concentration of less than 10 μg g^-1^, and Ni and Pb at a concentration of less than 30 μg g^-1^. Fe (490 μg g^-1^), Se (145 μg g^-1^), and Ag (1572 μg g^-1^) are significantly higher in this sample than in the other two groups. This pattern of depletion in most elements but enrichment in Se and Ag seemingly aligns with the reported mineral assemblages of Cu-Ag deposits formed within the Deweras group of the Magondi Belt [38, 72], including the modern mines at Mhangura (formerly Mangula) and Norah. The Mhangura and Norah deposits appear to be the leading candidates because they 1) host uranium minerals, which could contribute to a radiogenic lead isotope signature, 2) have economic silver mineralization, and 3) have economic concentrations of selenium [72, p. 126]. The hierarchical cluster analysis supports our hypothesis based on lead isotopes and chemistry by placing the Kent Estates HXR ingot in a distinct cluster (the “Phalaborwa and Magondi Belt” cluster) because of the amount of Se and Ag in this sample (see **Figs A and B in the S1 Appendix**).

## Discussion

**Fig 11:**
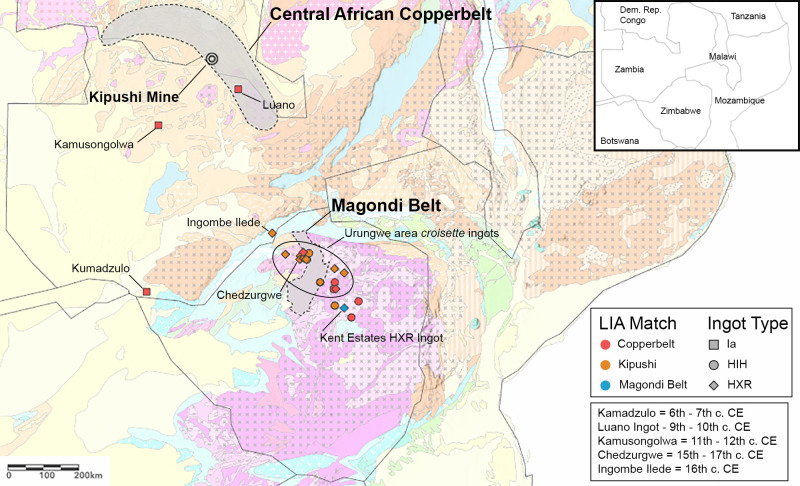
Map of the inferred provenance conclusion for each rectangular, fishtail, and *croisette* ingot in this study. Provenance results indicate that objects travelled significant distances to reach certain destinations and that interactions between the Copperbelt and areas further south can be traced back to the 6^th^-7^th^ century CE. Geological basemap adapted from Thiéblemont et al. [20].

The results from our lead isotopic and chemical analysis of 33 rectangular, fishtail, and *croisette* ingots from southern Africa establishes that there were three centers of *croisette* ingot production: the Central African Copperbelt, the Kipushi deposit, and the Magondi Belt (**Fig 11**). Object chemistry and isotopic data for these samples generally agree with deposit geochemistry and isotopic range, and suggest either that recycling was not a common practice in the production of large copper ingots, or that recycling activities were not based on mixing copper from various sources. Rademakers et al. [24] also concluded this in their study of *croisette* ingots from the Upemba Depression and western Copperbelt.

### Archaeological evidence: Central African Copperbelt

Sixteen samples exhibit characteristics that allow us to establish a provenance match to Cu-Co(-U) deposits within the Copperbelt. These samples are similar to copper samples from the Upemba Depression [24] and Botswana [33] which have previously been attributed to Copperbelt ores, and almost all have radiogenic lead isotopic data which forms a linear distribution on the ^206^Pb/^204^Pb vs ^207^Pb/^204^Pb plot that matches both the isochron age and overall patterning for Cu-Co(-U) Copperbelt ore samples (**Fig 8**). Furthermore, these samples have ^208^Pb/^204^Pb ratios that fall between 37 and 42, agreeing with the range exhibited by Copperbelt ore samples. Samples assigned to this provenance match in this study, from the Upemba Depression [24], and from the Tsodilo Hills [33] are also depleted in chalcophile elements but show relative enrichment in the siderophile elements Co and Ni, matching the overall geochemical profile of Cu-Co(-U) deposits in the Copperbelt. Unfortunately, the geochemical homogeneity of Cu-Co(-U) deposits in the Copperbelt precludes a more specific provenance assessment at this time.

To date, no molds for rectangular or fishtail ingots have been recorded on the Copperbelt, but many *croisette* ingot molds have been recovered in this area, along with extensive evidence for precolonial mining [42, 46]. Over 100 precolonial mines were documented in Katanga and Zambia by 1906. Most were in the Katangan Copperbelt (DRC) in an arc from Kolwezi to Kipushi [1, 2], but others were in the Zambian Copperbelt, the Kafue Hook, and the Domes region [1, 2].

The 16 samples reported here include all three recorded rectangular and fishtail ingots from Zambia (all dating before the 12^th^ century), an HIH ingot from the Harare tradition site of Graniteside, a 15^th^-17^th^ century cal CE HXR ingot from burial 8 of Ingombe Ilede, 10 HIH and HXR ingots from farms and towns in northern Zimbabwe, and the experimental “X” ingot. Our results illustrate that these ingots traveled significant distances from the Copperbelt to their sites of deposition (**Fig 11**). The Kumadzulo ingot (6^th^ or 7^th^ century) moved at least 600 km. HIH and HXR *croisette* ingots with Copperbelt lead isotopic and chemical signatures were found as far south as Harare, about the same distance from the Copperbelt. Their distribution implies a direct connection between Ingombe Ilede and the Copperbelt, as well as a connection between the Ingombe Ilede culture and sites of the contemporary Harare and Musengezi traditions of northern Zimbabwe.

### Archaeological evidence: Kipushi

The 16 ingots matched to the Zn-Pb-Cu deposit at Kipushi all have lead isotopic ratios tightly clustered around 18.05 in ^206^Pb/^204^Pb, 15.64 in ^207^Pb/^204^Pb, and 37.64 in ^208^Pb/^204^Pb. Although lead isotopic ratios on geological ores from Kipushi are tightly clustered and non-radiogenic [36, 73, 74] (**Fig 9**), the ratios of these ingots cluster even more tightly within the isotopic space of Kipushi ore samples (**Fig 8**). We assume that this subcluster represents the isotopic space of the precolonial mine in the oxidized surface deposits. One fragment of a casting spill excavated from furnaces beside the Kafue River, just across the border in Zambia, and one fragment of malachite recovered from the surface near these furnaces [2], also fall within this isotopic sub-cluster (**Fig 9**).

These ingots and smelted copper are highly enriched in Zn, As, Ag, Sb, and Pb when compared to the Copperbelt cluster, as is clearly seen in the boxplot in **Fig 10**. This plot also shows that these samples have lower concentrations of Co+Ni than samples attributed to the Copperbelt group (see above). The five chalcophile elements Zn, As, Ag, Sb, and Pb are known to be abundant at Kipushi, either as impurities substituted into the mineral lattice of malachite or in a range of copper arsenate, carbonate, oxide, phosphate, sulfate, vanadate, and chloride minerals that form in the supergene zone of this deposit [32, 37, p. 134–135]. The 16 ingots matching Kipushi also have strikingly similar chemical and isotopic data to three large HH ingots from the Upemba Depression that were analyzed by Rakemakers et al. [24]; these too clearly derive from Kipushi ores.

Archaeological sites in the vicinity of the Kipushi deposit include at least 57 discrete smelting sites with slag heaps, two large habitation sites, one campsite, and 71 individual *croisette* molds (for both HIH and HXR ingots). All were found on the Zambian side of the border with DRC [2]. Radiocarbon samples from Bisson’s excavations show activity at this mine as early as the 9^th^ century cal CE and may suggest an increase in production around the 14^th^ century cal CE [2], corresponding with the first appearance of HIH ingots in cemeteries of the Upemba Depression and in the archaeological record of Zimbabwe [51].

Of the 16 ingots matching the Kipushi ores, three are undated HIH ingots from northern Zimbabwe, two are 15^th^-17^th^ century cal CE HXR ingots from Ingombe Ilede (one from burial 2 and one from burial 8), two are HXR ingots from the 16^th^ century cal CE site of Chedzurgwe, and nine are surface HXR ingot finds distributed across northern Zimbabwe. These ingots were transported 500 km from Kipushi to Ingombe Ilede, and as much as 780 km for finds near Harare, Zimbabwe (**Fig 11**).

One interesting trend also observed in this dataset is the distinct change in copper source for HXR ingots as compared to the HIH ingot assemblage. 15 of 18 HIH ingots source to the Copperbelt while only 3 source to Kipushi. For HXR ingots 4 of 18 source to the Copperbelt and 13 of 18 to Kipushi. Taken together with Bisson’s [2] evidence for an increase in the exploitation of the Kipushi deposit in the 14^th^ century and the higher volume of HXR ingot mold fragments, it appears that Kipushi became a major hub for *croisette* ingot production around this time, though our understanding of who was exploiting the Kipushi deposit in the 14^th^ century is poor. We will further consider the archaeological implications of these data in a companion article.

### Archaeological evidence: Magondi Belt

One HXR ingot (from Kent Estates) does not match the Copperbelt or Kipushi in either lead isotopes or chemistry. The best match at present on lead isotope ratios is to ores of the Magondi Belt. We acknowledge that we require more lead isotopic data on ores from this district, but on present evidence there appears to be a trend line that is distinct from the Copperbelt ore data (**Fig 8**). Multivariate analysis of trace elements also places this ingot in a different group (the “Phalaborwa and Magondi Belt” cluster), distinct from the Kipushi ingots (the “Kipushi” cluster) and Copperbelt ingots (the “Copperbelt group 1” and “Copperbelt group 2” clusters) (**Figs A and B in the S1 Appendix**). It is also enriched in Ag and Se, both of which occur in economic concentrations in the Mhangura and Norah deposits [38, 72] in the Magondi Belt.

Evidence for precolonial mining in the Magondi Belt is substantial, particularly at the Alaska, Angwa, Mhangura, Norah, and Silverside mines [1, 3, 6] but has not received much archaeological attention. An HXR ingot mold was recovered from Golden Mile Mine, roughly 20 km away from the Mhangura mine in Zimbabwe. All of these lines of evidence suggest that the Kent Estates HXR ingot has a form copied from an ingot made on the Copperbelt or at Kipushi, but that the copper was mined locally somewhere within the Magondi Belt.

## Conclusion

Dozens of HIH and HXR copper ingots have been found in the Zambezi Valley and across the Zambezi plateau [51]. Since many of these finds are close to the copper deposits of the Magondi Belt of Northern Zimbabwe, Garlake [52] argued that most were made there. Subsequent excavations at Kipushi by Bisson [2] found and dated molds for both these ingot types, and more molds have been found at several other locations along the Copperbelt [46, Figs 2 and 3]. The alternative is therefore that these ingots were made along the border of Zambia and the DRC and traded from there to the Zambezi Valley and the Zimbabwe plateau.

We have analyzed 29 of the approximately 94 reported HIH and HXR ingots from Zambia and Zimbabwe and conclude that 28 of 29 derive from the Copperbelt, including 16 from the distinct Kipushi deposit within the Copperbelt (**Fig 11**). This work and our previous study [33] show that Copperbelt copper was transported to southwest Zambia (Kumadzulo) by the sixth or seventh century cal CE and to northwest Botswana by the eighth century cal CE. By the fourteenth century cal CE the flow of Copperbelt copper appears to have moved east to the middle Zambezi Valley (Ingombe Ilede) and the Urungwe District of northwest Zimbabwe, where sites like Chedzugwe have Ingombe Ilede pottery [52]. No HIH or HXR ingots have yet been reported from Botswana. The Kent Estates HXR ingot also demonstrates that there was at least some production of HXR ingots from Zimbabwe copper deposits (**Fig 11**).

This project is part of a larger ongoing study focused on the provenance of copper and copper-alloys in southern Africa. The samples presented in this paper represent 34 of the 277 copper, bronze, and brass samples that we were able to analyze from museums and institutions in southern Africa in 2019, and future works will continue to shed light on the hidden dynamics of interaction, migration, and exchange of copper and tin in southern Africa.

## Supporting information

S1 Appendix Additional sample selection and method details. (DOCX)

S2 Appendix Catalog of selected samples. (DOCX)

S3 Appendix Excel lead isotope database of relevant geological ore deposits. (XLSX)

S4 Appendix Inclusivity in global research questionnaire. (DOCX)
