# Supplementary material for: Reconstructing the geological provenance and long-distance movement of rectangular, fishtail, and croisette copper ingots in Iron Age Zambia and Zimbabwe
Source: PLoS One. 2023 Mar 22;18(3):e0282660. doi: 10.1371/journal.pone.0282660 (PMC10032518; doi:10.1371/journal.pone.0282660)
Supplement: S1 Appendix — (DOCX) [file pone.0282660.s001.docx]

## S1 Appendix

## Sample Selection

We selected a total of 33 rectangular, fishtail, *croisette* copper ingots (**Table 1**) from the Livingstone Museum in Livingstone, Zambia, the Museum of Human Sciences in Harare, Zimbabwe, and in the University of Cape Town archaeometallurgy study collection. Permit NHCC/SWR/05/008 was obtained from the National Heritage Conservation Committee in Zambia, and P 002/23 from the National Museums and Monuments board of Zimbabwe. The rectangular and fishtail ingots are from the sites of Kumadzulo (rectangular) [1], Kamusongolwa (fishtail) [2], and Luano (fishtail) [3]. The HIH ingots sampled for this study were primarily from farms and towns located in northern Zimbabwe [4], but also includes one object likely from the Graniteside site in Harare^^[[1]](#footnote-1)^^. HXR ingots sampled by this project include three of the eight Ingombe Ilede HXR ingots [5], two samples from the Chedzurgwe site^^[[2]](#footnote-2)^,^[[3]](#footnote-3)^^ [6], one sample from Kent estates, outside Harare, Zimbabwe, and 12 samples from farms and towns located in northern Zimbabwe [4]. The experimental “X” ingot was cast from copper ore mined from Kansanshi mine in the 1970’s and was given to the authors in 2016 by Dr. Mike Bisson.

## Data Processing

The measured lead isotopic ratios for these samples include both radiogenic lead and common lead values. Radiogenic lead refers to values which are above the average modern-day lead isotopic signature in the Earth’s crust (^206^Pb/^204^Pb = 18.700, ^207^Pb/^204^Pb = 15.628, ^208^Pb/^204^Pb = 38.63), while those below these values are referred to as common lead [7; 8]. For those samples with common lead ratios, we can calculate model age for the ore deposit using the MATLAB solver published by Albarède et al. [9]. However, for samples with radiogenic values, calculated model ages using the MATLAB model age solver are hundreds or thousands of millions of years into the future. This is because of the excess contribution of ^206^Pb, ^207^Pb, and ^208^Pb via the radioactive decay of uranium and thorium. For samples with these radiogenic values, it is sometimes the case that the ratios obtained from a single deposit or mining district define a linear array, or isochron, on the ^206^Pb/^204^Pb vs ^207^Pb/^204^Pb plot. This approach is common in geological applications of lead isotopes, as a single ore deposit can produce datapoints at varying stages along a linear distribution based on variable concentrations of U and Th in individual crystals of copper sulfide minerals at the time of their formation (T=0). Steeper slopes are more representative of older deposits while shallower slopes are representative of younger deposits. For samples in this article, isochron ages were calculated using the program isoplotR and the “three ratio” Pb-Pb isochron generator, which also produces an associated measure of error for the age, or MSWD [11]. This is a measure of the mean distance of datapoints from the regression line. The value of MSWD is important for geochemists interested in dating ore deposits and MSWD values much greater than 1 are typically referred to as “errorchrons”. This distinction is not of significance for archaeological provenance studies, as we are not interested in dating episodes of mineral formation and cannot assume that ores used to cast archaeological objects came from a single mine. Lead isotopic data was also compared to our geological database consisting of 752 lead isotopic datapoints on sulfide and copper carbonate ore samples from varying deposits and districts in southern Africa.

Concentrations of elements were log-transformed for both descriptive and multivariate statistical analysis, including hierarchical cluster analysis and principal component analysis. The hierarchical cluster analysis was performed using the *hcut* function in the factoextra R package, using the ward.D agglomeration method and euclidean distance to calculate the dissimilarity matrix. Results were then visualized using the *fviz_dend* function in the factoextra R package. Groups were investigated by PCA, using the *prcomp* function in the stats R package, to assess variation within the hierarchical cluster analysis relating to source attribution, technology, and deposit geochemistry. Missing values for these multivariate statistics can be handled in a number of different ways. We chose the substitution method for handling these cases because we knew that all missing values fell below the detection limit of the Elan DRC-II ICP-MS instrument. Therefore, these values must be near-zero and we used the value “0.001” for these cases.

We used the gap statistics method in the function *fviz_nbclust* from the factoextra R package to determine the optimum number of clusters in our dataset. This algorithm works by comparing the total intra-cluster variation against uniformly randomized data in order to assess the optimal number of clusters to create a data structure as far away from the random data as possible [12]. This statistical measure determined that the optimal number of clusters in our dataset was eight. However, upon assessing these groupings, we determined that two clusters were subgroupings of others, and therefore reduced the number of distinct clusters to six.

## Hierarchical Cluster Analysis of Sample Chemistry

Multivariate statistical groupings were produced from the entire ingot assemblage, including 12 bar, bun, lerale, musuku, and nail head ingots which will published in a future paper. The multivariate groupings were assessed independently from the ingot provenance hypotheses in the main text, yet multivariate groupings correlate with these attributions. These groupings can be defined from left to right on the hierarchical cluster analysis dendrogram (**Fig. A**) as: 1) “Copperbelt group 1”, 2) “Phalaborwa and Magondi Belt”, 3) “Copperbelt group 2”, 4) “Kipushi”, 5) “Phalaborwa and Copper Queen”, and 6) “Phalaborwa and other”. This paper will focus on clusters 1, 2, 3, and 4, while further data from clusters 2, 5, and 6 will be discussed in a future paper.

Clusters 1-3 are depleted in chalcophile elements (Zn, As, Se, Ag, and Pb) and in Fe and therefore fall on the left side of the cluster analysis dendrogram (**Figs A and B**). The distinction between “Copperbelt groups” 1 and 2 lies in differences in Cr, Fe, Se, and Ag concentration; notably, “Copperbelt group 1” has Cr and Se values above detection limits and a higher average Fe concentration (67 ppm vs 24 ppm for Copperbelt group 2”) (**Fig. B**). The gap statistical procedure initially separated the fishtail ingot Zim-Luano-4, the HXR ingot Zim-ZMHS-22, and the HIH ingot Zim-ZMHS-24 from the remaining samples in the “Copperbelt group 2” cluster, but most of their chemical values fall within the range of variation for “Copperbelt group 2” samples. The only difference is in the amount of Sn, which is below detection limits for these three samples. We therefore kept them associated with “Copperbelt group 2” samples, all of which have low values of Sn (mean of 2.5 ppm).

The mixed cluster, “Phalaborwa and Magondi Belt”, is also similarly depleted in most elements, but exhibits higher average Fe (234 ppm), Se (85 ppm), and Ag (625 ppm) values (**Fig. B**). The patterning of Fe, As, Se, and Ag in samples within this mixed group suggests different deposits, and the HXR ingot from Kent estates (Zim-Kent-1) has more Ag than Se, an Fe value lower than its Ag values, and a low As value. This patterning is consistent with the published ore mineral assemblages of copper mines in the Magondi belt.

The “Kipushi” and “Phalaborwa and Copper Queen” clusters fall to the right of the cluster analysis dendrogram because they exhibit higher concentrations of Fe, Zn, As, Se, Ag, Sn, Sb, and Pb (**Figs A and B**). However, the “Kipushi” cluster exhibits lower average concentrations of chalcophile elements than “Phalaborwa and Copper Queen (Zn: 59 vs 494 ppm, As: 869 vs 2929 ppm, Se: 8 vs 117 ppm, Sn: 3 vs 214 ppm, Sb: 28 vs 150 ppm, and Pb: 378 vs 2297 ppm). The same division is seen in siderophile concentrations (Fe: 27 ppm vs 8396 ppm, Co: <1 vs 99 ppm, Ni: 2 vs 708 ppm, and Mo: all but one below instrument detection limits vs 0.5 ppm), but higher average Ag (1254 vs 206 ppm) (**Fig. B**).

These groups defined by hierarchical cluster analysis broadly agree with those we identified in our assessment of the lead isotopic and chemical data. Therefore, we are confident that these groups are valid and indicative of the initial geological source for the copper used in the production of these objects.

## S1 References

1. Vogel JO. Kamadzulo: an Early Iron Age Village Site in southern Zambia. (No. 3). National Museums of Zambia by Oxford University Press; 1971.
2. Daniels S. A note on the Iron Age material from Kamusongolwa Kopje, Zambia. The South African Archaeological Bulletin. 1967; 22(88): 142-150.
3. Bisson MS. Precolonial copper metallurgy: sociopolitical context. In: Vogel, J.O. (Ed.), Ancient African Metallurgy: the Sociocultural Context. Altamira Press, Walnut Creek; 2000. pp. 83-146.
4. Swan LM. Economic and ideological roles of copper ingots in prehistoric Zimbabwe. Antiquity. 2007; 81: 999-1012.
5. Fagan BM, Phillipson DW, Daniels SGH. Iron Age cultures in Zambia, volume II: Dambwa, Ingombe Ilede, and the Tonga. London: Chatto & Windus; 1969.
6. Garlake P. Iron Age sites in the Urungwe District of Rhodesia. South African Archaeological Bulletin. 1970; 25: 25-44. <https://doi.org/10.2307/3888765>
7. Faure G, Mensing TM. Principles and applications. John Wiley & Sons, Inc; 2005.
8. Stacey JS, Kramers JD. Approximation of terrestrial lead isotope evolution by a two-stage model. Earth Planet Sci. Lett. 1975; 26: 207-221. <https://doi.org/10.1016/0012-821X(75)900886>
9. Albarède F, Desaulty AM, Blichert-Toft J. A geological perspective on the use of Pb isotopes in archaeometry. Archaeometry. 2012; 54: 853-867. <https://doi.org/10.1111/j.1475-4754.2011.00653.x>
10. Goodall E. Report on an ancient burial ground, Salisbury Southern Rhodesia. Musée royal de l'Afrique centrale, Annales in 8, Sciences géologiques. 1962; 40: 315-322.
11. Vermeesch P. IsoplotR: a free and open toolbox for geochronology. Geosci. Front. 2018; 9: 1479-1493. <https://doi.org/10.1016/j.gsf.2018.04.001>.
12. Tibshirani R, Walther G, Hastie T. Estimating the number of clusters in a data set via the gap statistic. Journal of the Royal Statistical Society: Series B (Statistical Methodology). 2001; 63(2): 411-423. <https://doi.org/10.1111/1467-9868.00293>
13. Thiéblemont D, Liégeois JP, Fernandez-Alonso M, Ouabadi A, Le Gall B, Maury R, Jalludin M, Vidal M, Ouattara Gbélé C, Tchaméni R, Michard A, Nehlig P, Rossi P, Frédéric C. Geological Map of Africa at 1:10 M scale. CGMW-BRGM; 2016. <https://doi.org/10.14682/2016GEOAFR>.

## S1 Figures


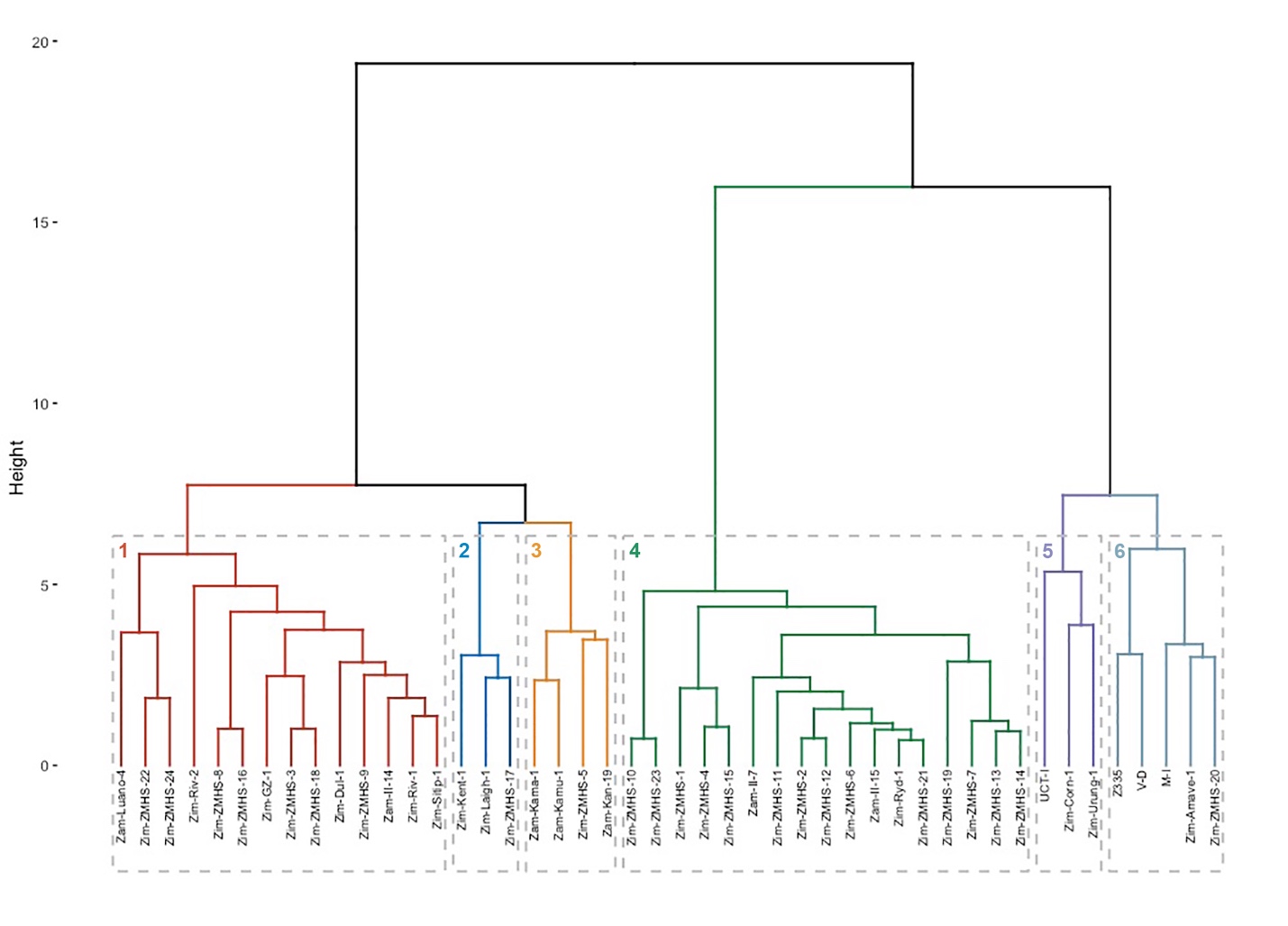


**Fig. A**: Heirarchical clustering dendrogram of the total ingot chemistry assemblage, including bun, bar, lerale, and musuku ingot types. Groups are defined from left to right as: 1) “Copperbelt group 1”, 2) “Phalaborwa and Magondi Belt”, 3) “Copperbelt group 2”, 4) “Kipushi”, 5) “Phalaborwa and Copper Queen”, and 6) “Phalaborwa and Other”.


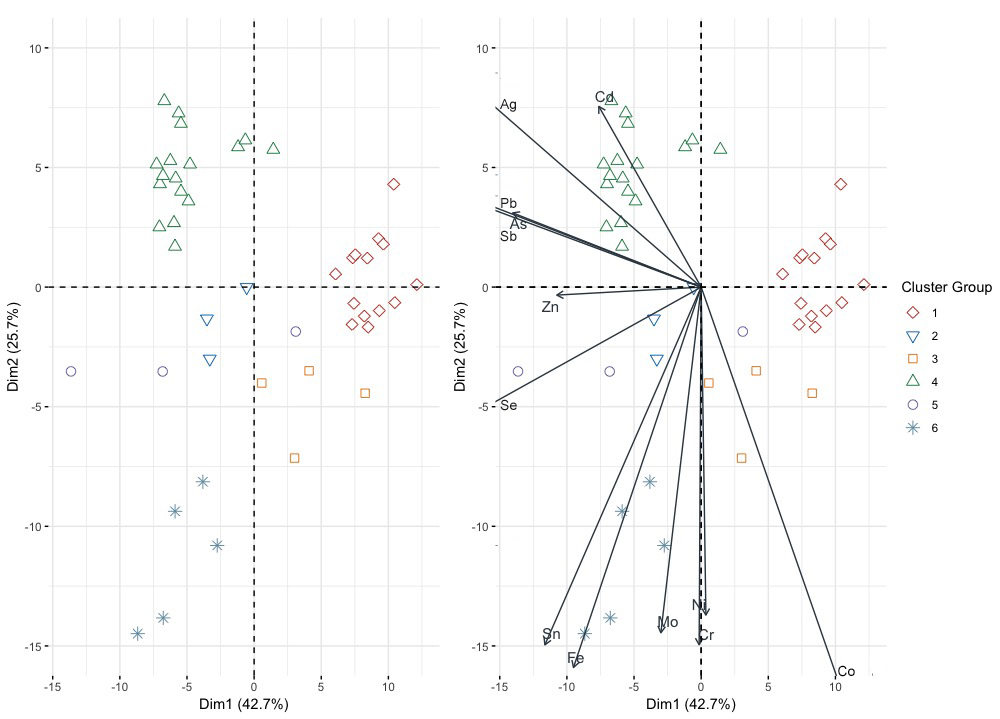


**Fig. B:** Biplots of PCA1 (42.7% of total variance) versus PCA2 (25.7% of total variance) and elemental contribution to variance.


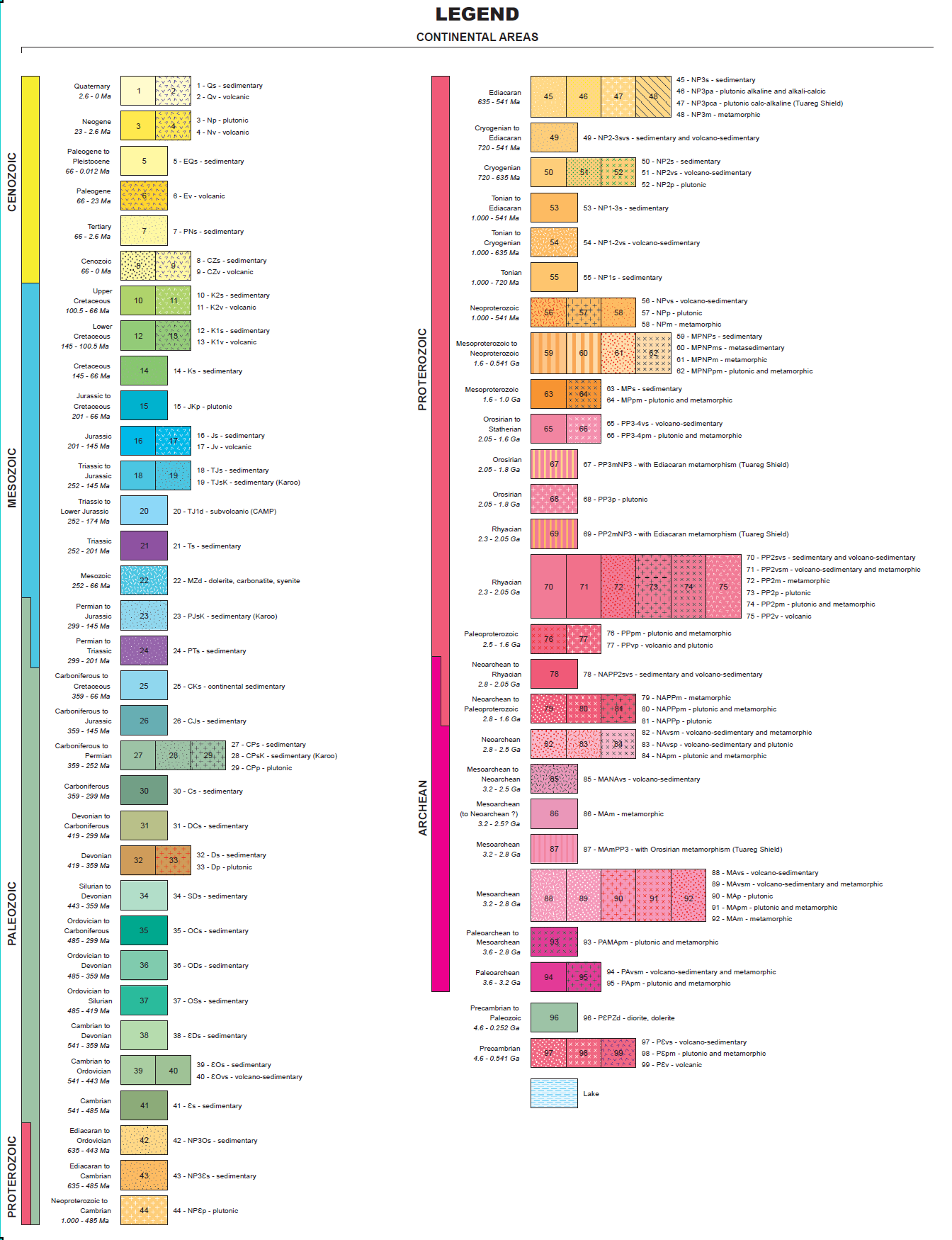


**Fig. C:** Legend for the geological basemap used in ​**Figs 1, 3, and 11**​ of the main text. Adapted from Thiéblemont et al. [13].

1. The label for this sample is recorded as “Graniteside. 1731-CC-3?”, matching both the name and site number for the Harare period Graniteside burial site [10] despite the question mark. [↑](#footnote-ref-1)
2. One sample (Zim-Ryd-1) from Chedzurgwe was a separately stored cutoff that had been previously removed from an HXR ingot and stored with the Chedzurgwe excavation materials. At the time of documentation, we did not know that this was a subsample of sample Zim-ZMHS-2 and only realized the duplication during the course of laboratory work. Therefore, we have sampled 33 individual ingots, but present data on 34 samples. [↑](#footnote-ref-2)
3. Samples from Chedzurgwe were also labeled as Rydings, however we will use the site name Chedzurgwe in this publication. [↑](#footnote-ref-3)
