# Supplementary material for: Reconstructing the geological provenance and long-distance movement of rectangular, fishtail, and croisette copper ingots in Iron Age Zambia and Zimbabwe
Source: PLoS One. 2023 Mar 22;18(3):e0282660. doi: 10.1371/journal.pone.0282660 (PMC10032518; doi:10.1371/journal.pone.0282660)

## S2 Appendix

## Object Catalog

| Sample ID | Zim-Dul-1 |
| --- | --- |
| Ingot Type | HIH |
| Country | Zimbabwe |
| Site | Dunlorne Farm |
| Museum Site Reference | 1730 CB 7 |
| Cultural Association | - |
| Context | - |
| Housing Museum | Museum of Human Sciences, Harare |
| Museum Accession Number | - |
| Reference | - |
| Weight (g) | 2129 |
| Length (cm) | 22 |


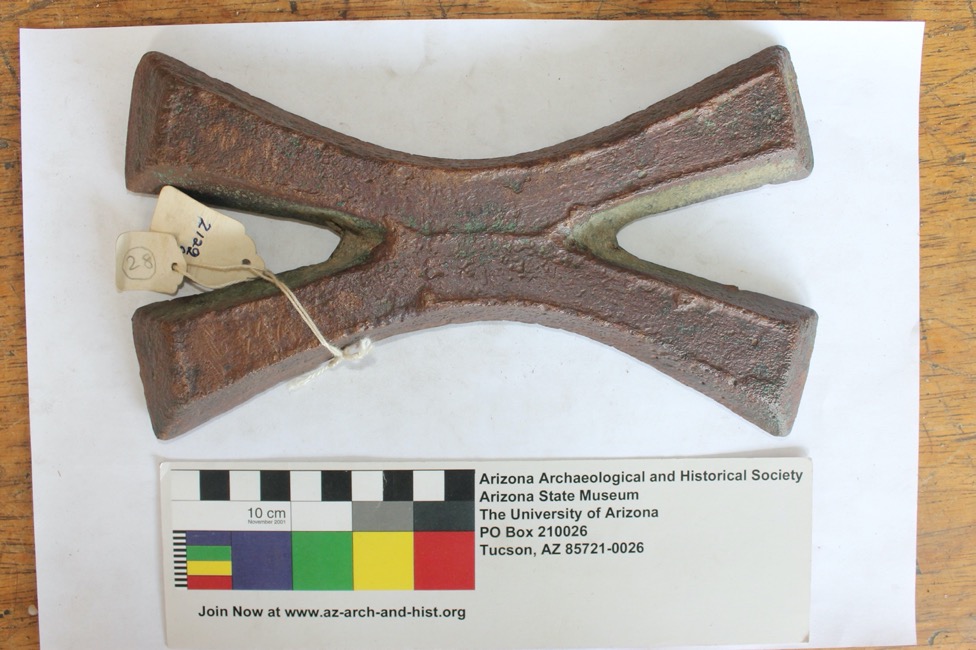


| Sample ID | Zim-Kent-1 |
| --- | --- |
| Ingot Type | HXR |
| Country | Zimbabwe |
| Site | Kent Estates |
| Museum Site Reference | 1730 DC 22 |
| Cultural Association | - |
| Context | - |
| Housing Museum | Museum of Human Sciences, Harare |
| Museum Accession Number | - |
| Reference | - |
| Weight (g) | - (Partial Ingot) |
| Length (cm) | Estimated 32cm (Partial Ingot) |


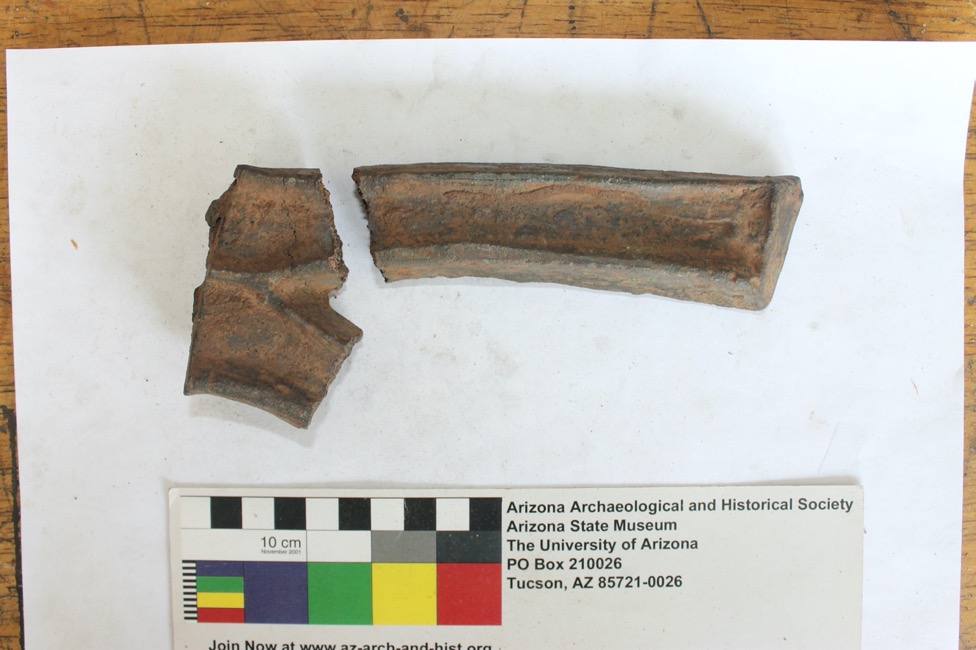


| Sample ID | Zim-Riv-1 |
| --- | --- |
| Ingot Type | HIH |
| Country | Zimbabwe |
| Site | Riverside Farm |
| Museum Site Reference | 1730 CB 33 |
| Cultural Association | - |
| Context | - |
| Housing Museum | Museum of Human Sciences, Harare |
| Museum Accession Number | - |
| Reference | - |
| Weight (g) | - |
| Length (cm) | 18 |


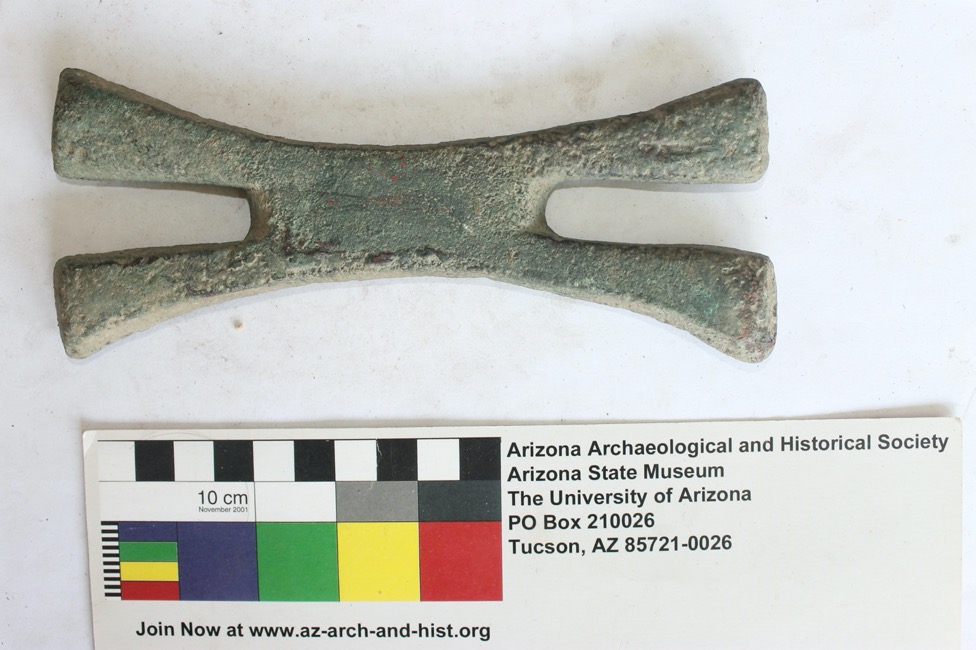


| Sample ID | Zim-Riv-2 |
| --- | --- |
| Ingot Type | HIH |
| Country | Zimbabwe |
| Site | Riverside Farm |
| Museum Site Reference | 1730 CB 33 |
| Cultural Association | - |
| Context | - |
| Housing Museum | Museum of Human Sciences, Harare |
| Museum Accession Number | - |
| Reference | - |
| Weight (g) | - |
| Length (cm) | 19 |


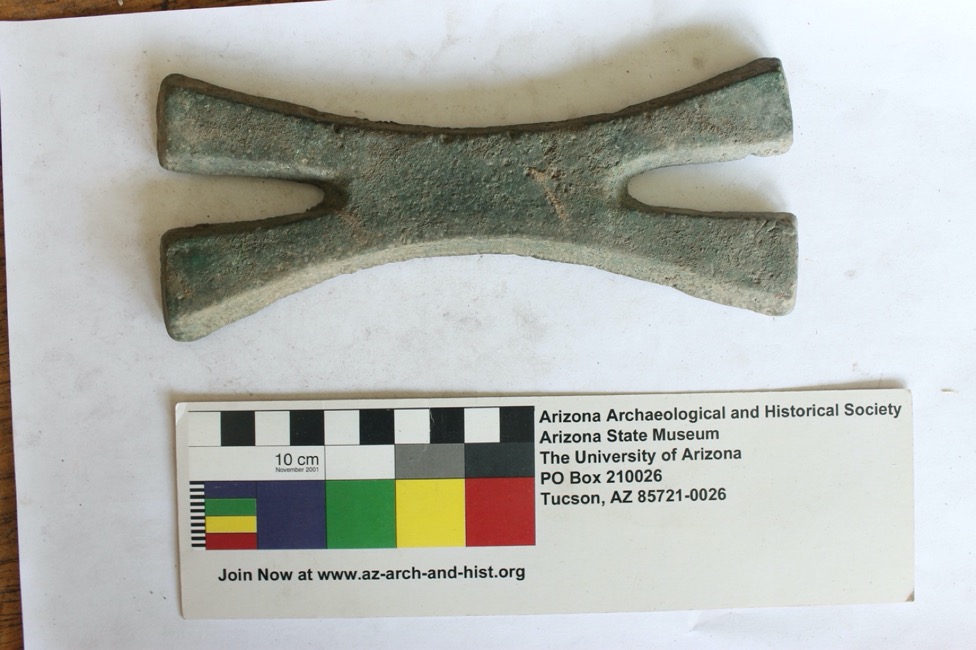


| Sample ID | Zim-ZMHS-1 |
| --- | --- |
| Ingot Type | HXR |
| Country | Zimbabwe |
| Site | Yeadon Farms |
| Museum Site Reference | 1629 DA 6 |
| Cultural Association | - |
| Context | - |
| Housing Museum | Museum of Human Sciences, Harare |
| Museum Accession Number | QMIA 4461 |
| Reference | Swan 2007 |
| Weight (g) | 4307 |
| Length (cm) | 31.5 |


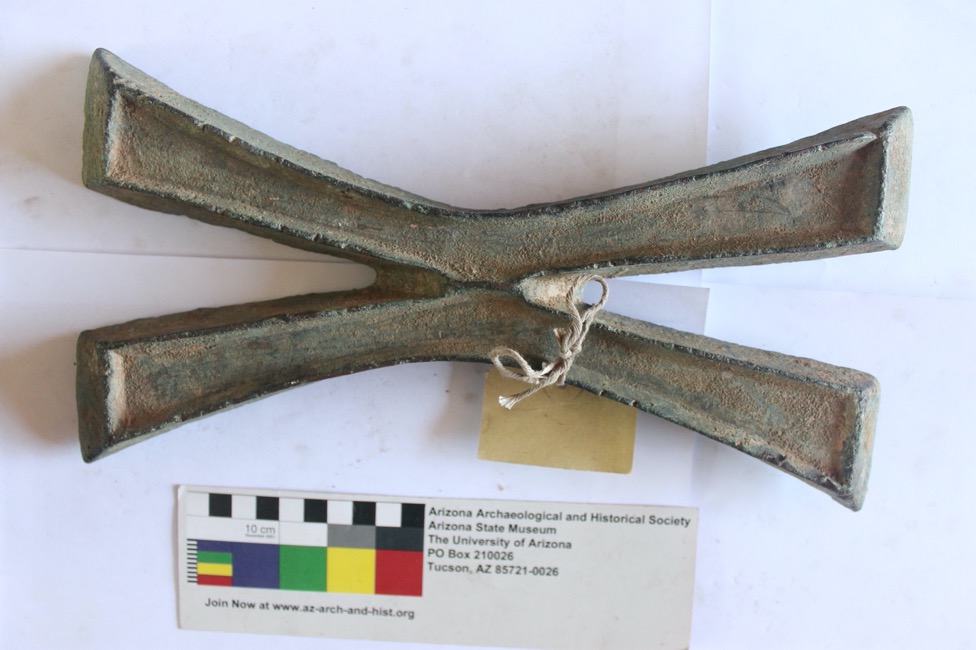


| Sample ID | Zim-ZMHS-2, Zim-Ryd-1* |
| --- | --- |
| Ingot Type | HXR |
| Country | Zimbabwe |
| Site | Chedzurgwe (Rydings) |
| Museum Site Reference | 1629 DC 5 |
| Cultural Association | Ingombe Ilede |
| Context | Surface |
| Housing Museum | Museum of Human Sciences, Harare |
| Museum Accession Number | QMIA 4462 |
| Reference | Garlake 1970 |
| Weight (g) | 3490 |
| Length (cm) | 29.5 |

*Zim-Ryd-1 is a duplicate sample of Zim-ZMHS-2 that was stored at the Museum of Human Sciences as a separate cutoff.


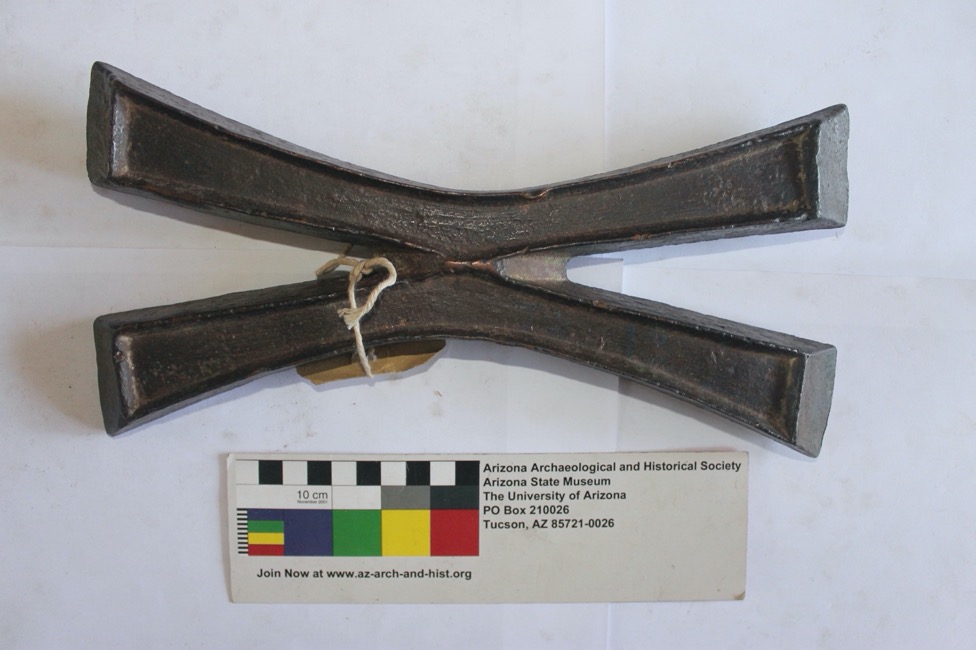


| Sample ID | Zim-ZMHS-4 |
| --- | --- |
| Ingot Type | HIH |
| Country | Zimbabwe |
| Site | Karoi area |
| Museum Site Reference | - |
| Cultural Association | - |
| Context | - |
| Housing Museum | Museum of Human Sciences, Harare |
| Museum Accession Number | QMIA 4464 |
| Reference | Swan 2007 |
| Weight (g) | 679 |
| Length (cm) | 18.5 |


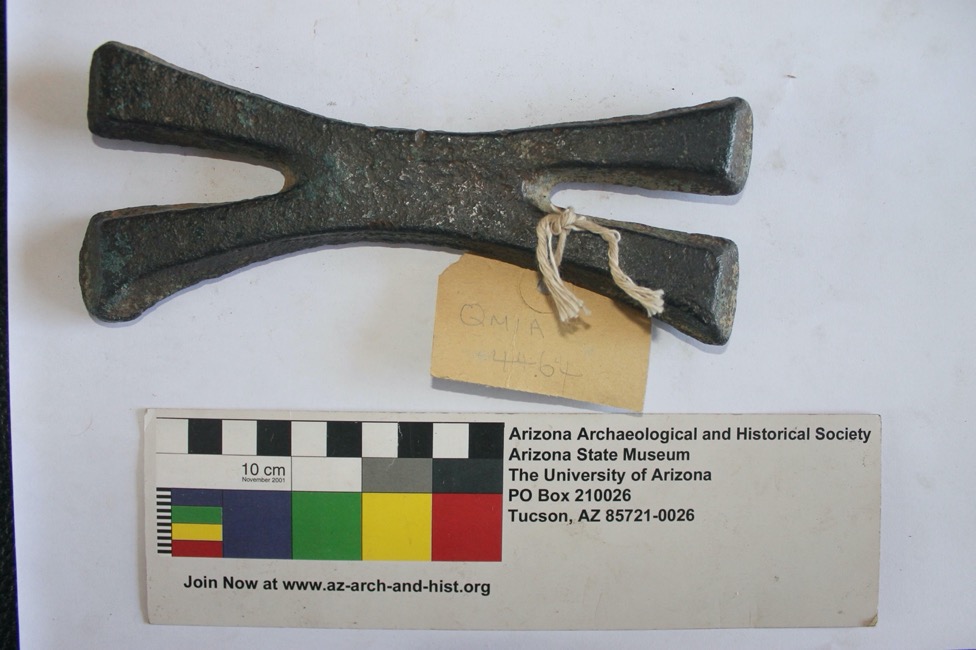


| Sample ID | Zim-ZMHS-5 |
| --- | --- |
| Ingot Type | HXR |
| Country | Zimbabwe |
| Site | Unknown |
| Museum Site Reference |  |
| Cultural Association |  |
| Context |  |
| Housing Museum | Museum of Human Sciences, Harare |
| Museum Accession Number | QMIA 4463 |
| Reference | - |
| Weight (g) | - (Partial Ingot) |
| Length (cm) | Estimated 27cm (Partial Ingot) |


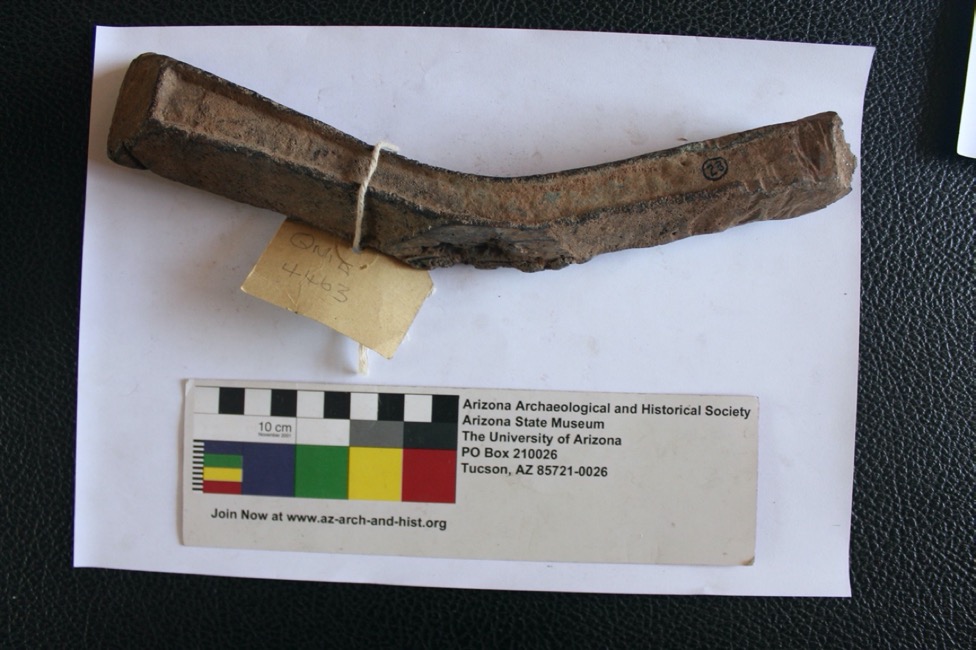


| Sample ID | Zim-ZMHS-6 |
| --- | --- |
| Ingot Type | HXR |
| Country | Zimbabwe |
| Site | Zave |
| Museum Site Reference | 1730 AA 8 |
| Cultural Association | - |
| Context | - |
| Housing Museum | Museum of Human Sciences, Harare |
| Museum Accession Number | QMIA 4455 |
| Reference | Swan 2007 |
| Weight (g) | - (Partial Ingot) |
| Length (cm) | Estimated 40cm (Partial Ingot) |


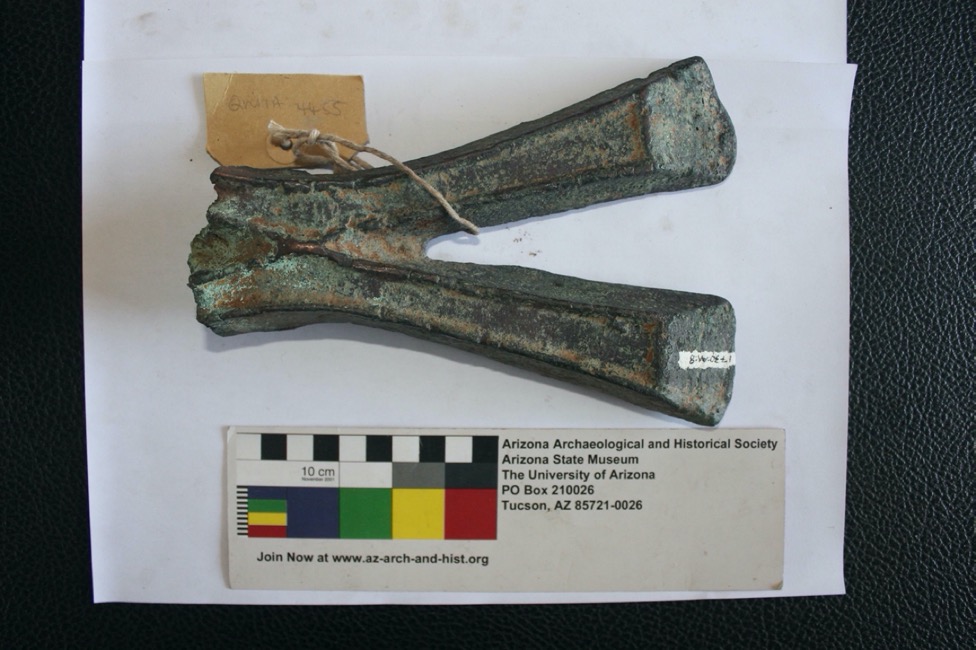


| Sample ID | Zim-ZMHS-7 |
| --- | --- |
| Ingot Type | HXR |
| Country | Zimbabwe |
| Site | Kashwao East |
| Museum Site Reference | 1730 BA 23 |
| Cultural Association | - |
| Context | - |
| Housing Museum | Museum of Human Sciences, Harare |
| Museum Accession Number | QMIA 4457 |
| Reference | Swan 2007 |
| Weight (g) | 3273 |
| Length (cm) | 29 |


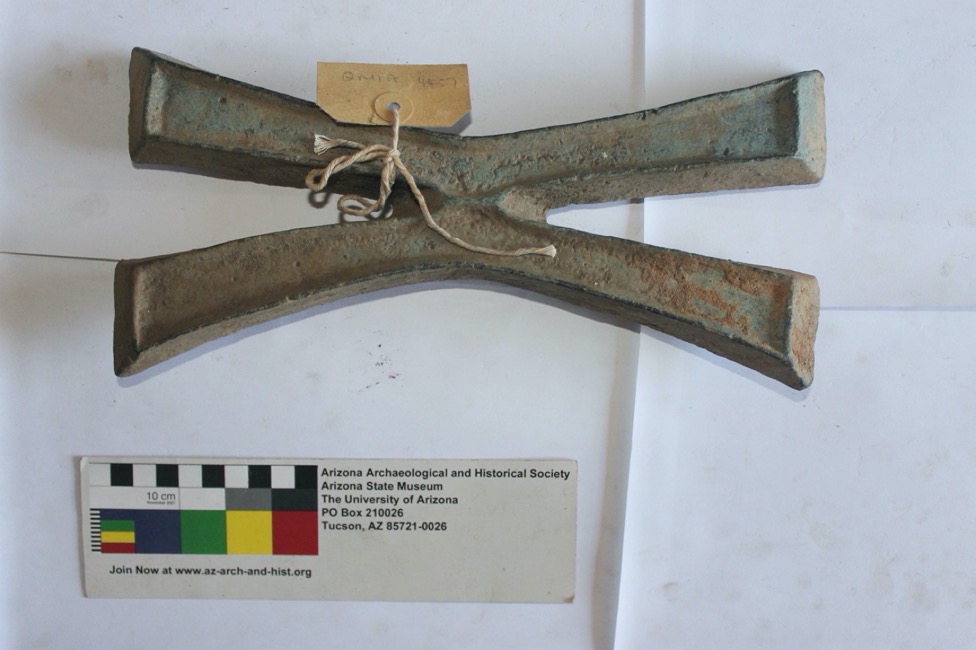


| Sample ID | Zim-ZMHS-8 |
| --- | --- |
| Ingot Type | HIH |
| Country | Zimbabwe |
| Site | Zave |
| Museum Site Reference | 1730 AA 8 |
| Cultural Association | - |
| Context | - |
| Housing Museum | Museum of Human Sciences, Harare |
| Museum Accession Number | QMIA 4456 |
| Reference | Swan 2007 |
| Weight (g) | 1125 |
| Length (cm) | 20.5 |


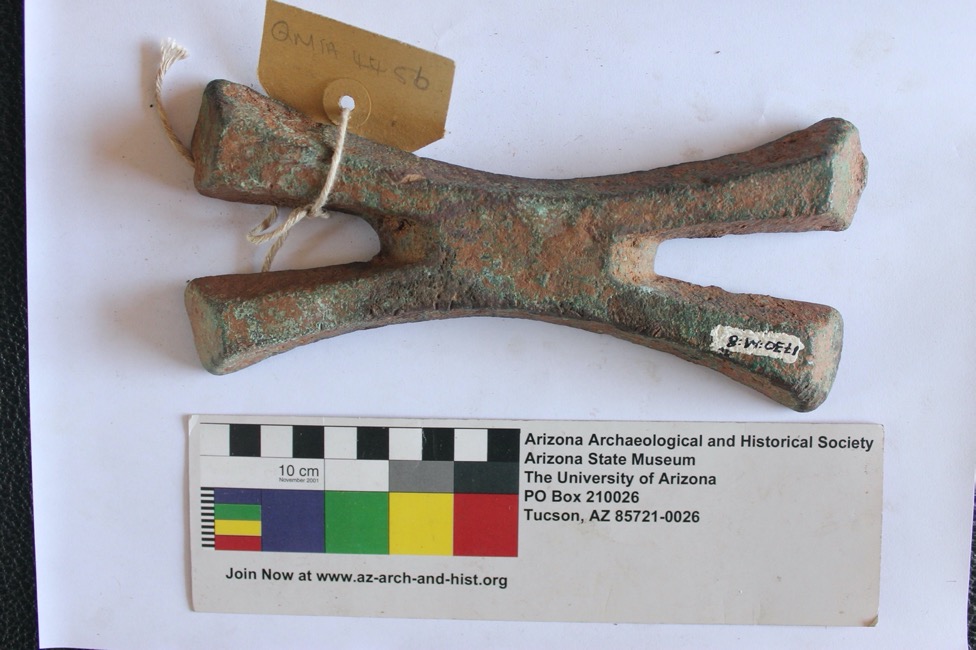


| Sample ID | Zim-ZMHS-9 |
| --- | --- |
| Ingot Type | HIH |
| Country | Zimbabwe |
| Site | Graniteside |
| Museum Site Reference | 1731 CC 3 |
| Cultural Association | Harare |
| Context | - |
| Housing Museum | Museum of Human Sciences, Harare |
| Museum Accession Number | QMIA 4459 |
| Reference | Swan 2007 |
| Weight (g) | 443 |
| Length (cm) | 17.4 |


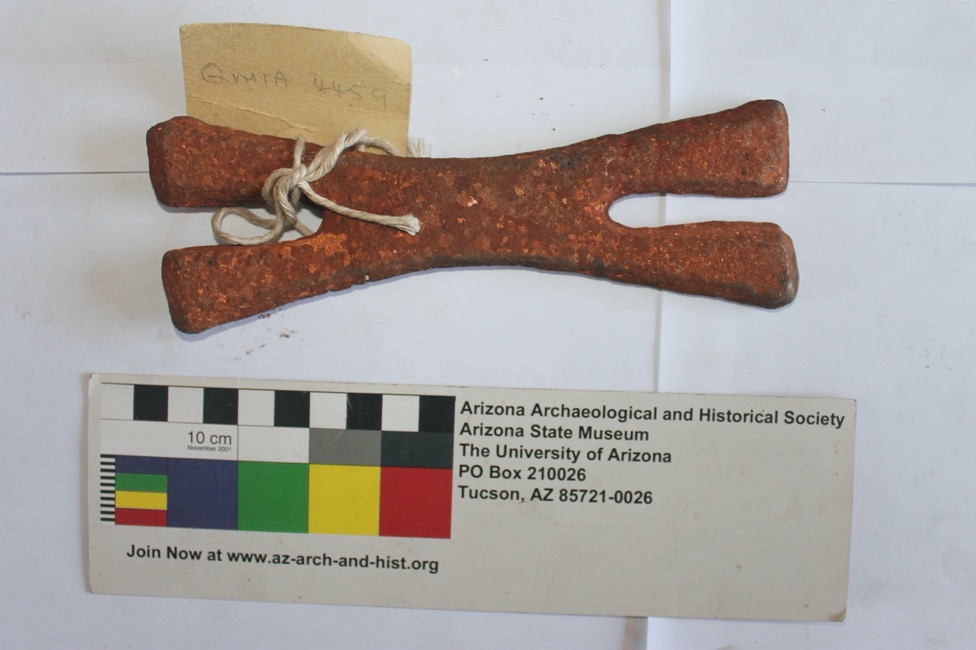


| Sample ID | Zim-ZMHS-10 |
| --- | --- |
| Ingot Type | HIH |
| Country | Zimbabwe |
| Site | Makwiro |
| Museum Site Reference | 1730 CD 10 |
| Cultural Association | - |
| Context | - |
| Housing Museum | Museum of Human Sciences, Harare |
| Museum Accession Number | QMIA 4458 |
| Reference | Swan 2007 |
| Weight (g) | 1114 |
| Length (cm) | 18.3 |


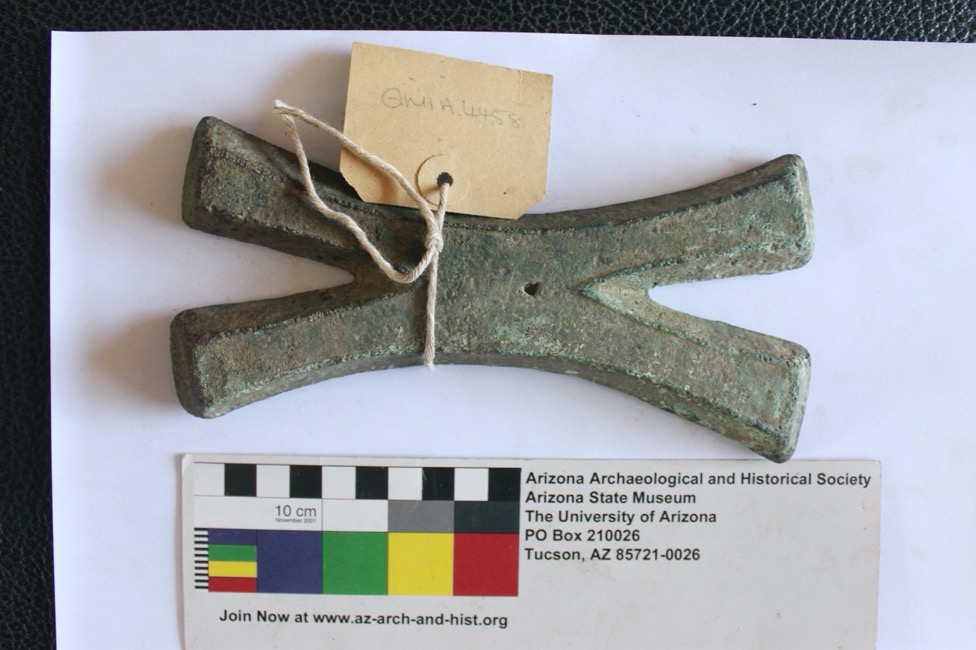


| Sample ID | Zim-ZMHS-11 |
| --- | --- |
| Ingot Type | HXR |
| Country | Zimbabwe |
| Site | Zave |
| Museum Site Reference | 1730 AA 8 |
| Cultural Association | - |
| Context | - |
| Housing Museum | Museum of Human Sciences, Harare |
| Museum Accession Number | QMIA 4454 |
| Reference | Swan 2007 |
| Weight (g) | 1709 (Partial Ingot) |
| Length (cm) | 19.9 (Partial Ingot) |


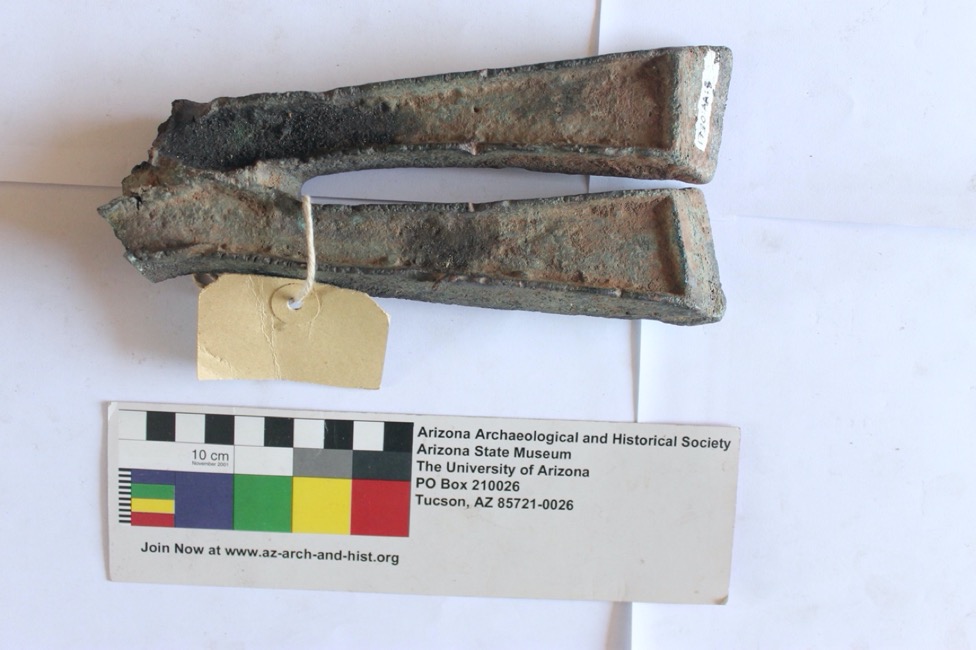


| Sample ID | Zim-ZMHS-12 |
| --- | --- |
| Ingot Type | HXR |
| Country | Zimbabwe |
| Site | Karoi Dixie farm |
| Museum Site Reference | 1629 DC 24 |
| Cultural Association | - |
| Context | - |
| Housing Museum | Museum of Human Sciences, Harare |
| Museum Accession Number | QMIA 3068 |
| Reference | Swan 2007 |
| Weight (g) | 3573 |
| Length (cm) | 31 |


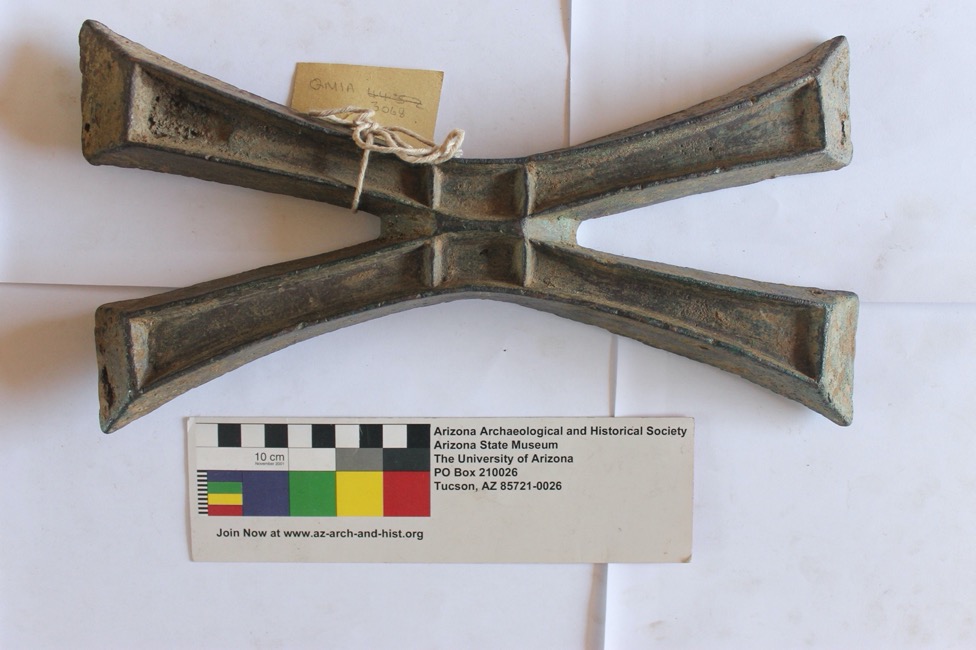


| Sample ID | Zim-ZMHS-13 |
| --- | --- |
| Ingot Type | HXR |
| Country | Zimbabwe |
| Site | Chedzurgwe (Rydings) |
| Museum Site Reference | 1629 DC 5 |
| Cultural Association | Ingombe Ilede |
| Context | Surface |
| Housing Museum | Museum of Human Sciences, Harare |
| Museum Accession Number | QMIA 4451 |
| Reference | Garlake 1970 |
| Weight (g) | 3068 |
| Length (cm) | 27.5 |


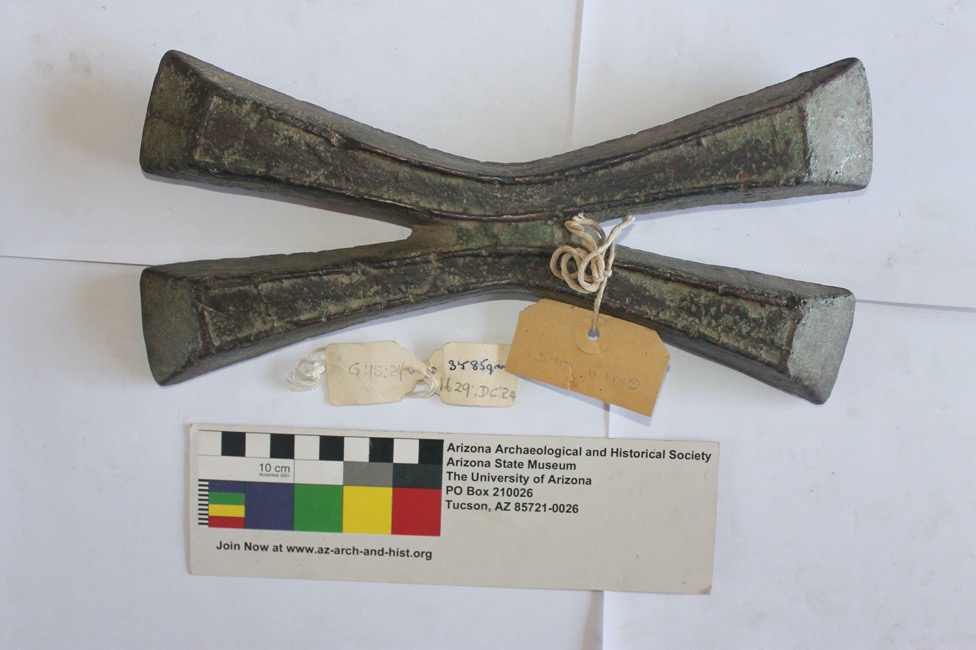


| Sample ID | Zim-ZMHS-14 |
| --- | --- |
| Ingot Type | HXR |
| Country | Zimbabwe |
| Site | Bassett Farm |
| Museum Site Reference | 1730 AB 3 |
| Cultural Association | - |
| Context | - |
| Housing Museum | Museum of Human Sciences, Harare |
| Museum Accession Number | QMIA 4453 |
| Reference | Swan 2007 |
| Weight (g) | 3205 |
| Length (cm) | 29.6 |


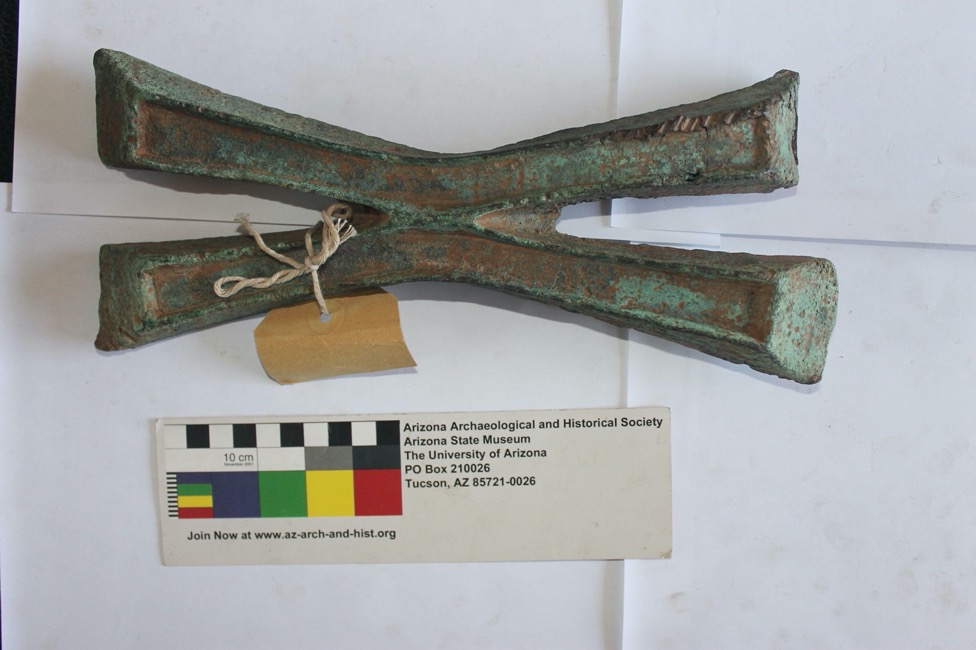


| Sample ID | Zim-ZMHS-15 |
| --- | --- |
| Ingot Type | HXR |
| Country | Zimbabwe |
| Site | Karoi area |
| Museum Site Reference | - |
| Cultural Association | - |
| Context | - |
| Housing Museum | Museum of Human Sciences, Harare |
| Museum Accession Number | QMIA 4467 |
| Reference | Swan 2007 |
| Weight (g) | 3347 |
| Length (cm) | 29.9 |


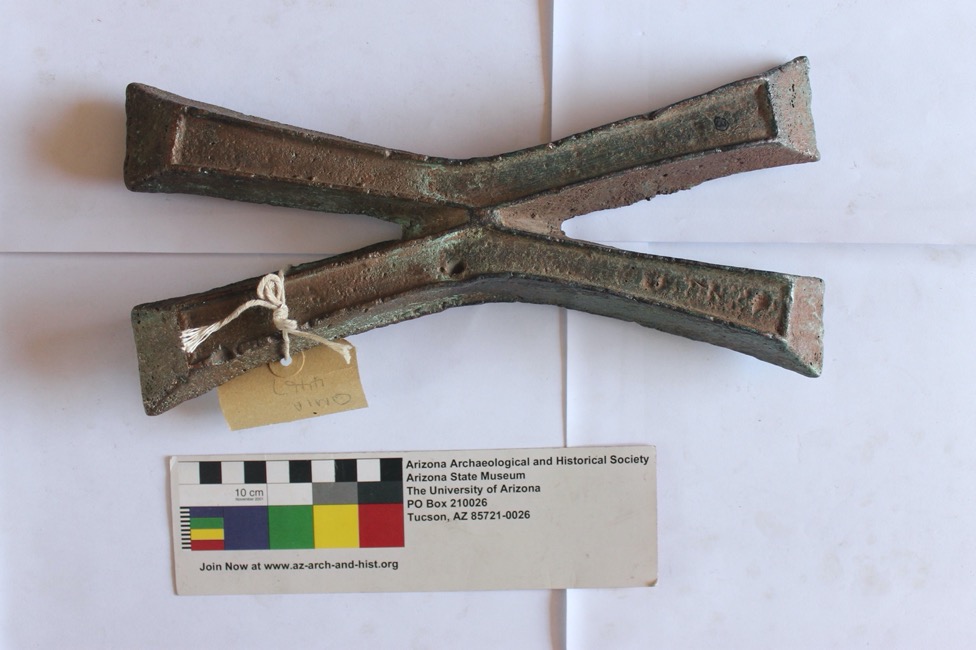


| Sample ID | Zim-ZMHS-16 |
| --- | --- |
| Ingot Type | HXR |
| Country | Zimbabwe |
| Site | Karoi area |
| Museum Site Reference | - |
| Cultural Association | - |
| Context | - |
| Housing Museum | Museum of Human Sciences, Harare |
| Museum Accession Number | QMIA 4465 |
| Reference | Swan 2007 |
| Weight (g) | 2967 |
| Length (cm) | 29 |


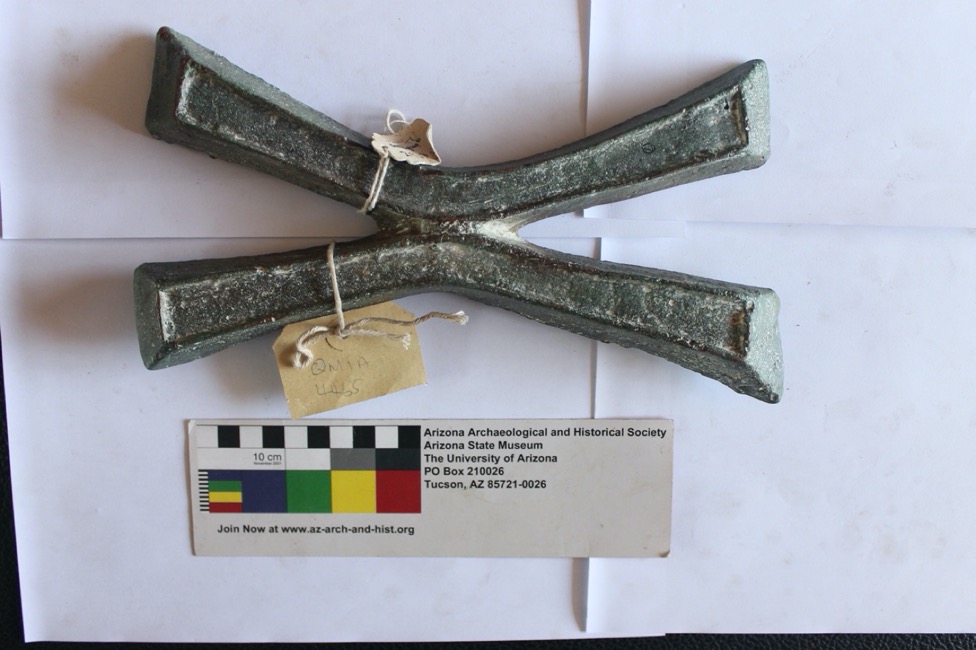


| Sample ID | Zim-ZMHS-18 |
| --- | --- |
| Ingot Type | HIH |
| Country | Zimbabwe |
| Site | Horizon Farm |
| Museum Site Reference | - |
| Cultural Association | - |
| Context | - |
| Housing Museum | Museum of Human Sciences, Harare |
| Museum Accession Number | QMIA 4498 |
| Reference | Swan 2007 |
| Weight (g) | 350 |
| Length (cm) | 17.4 |


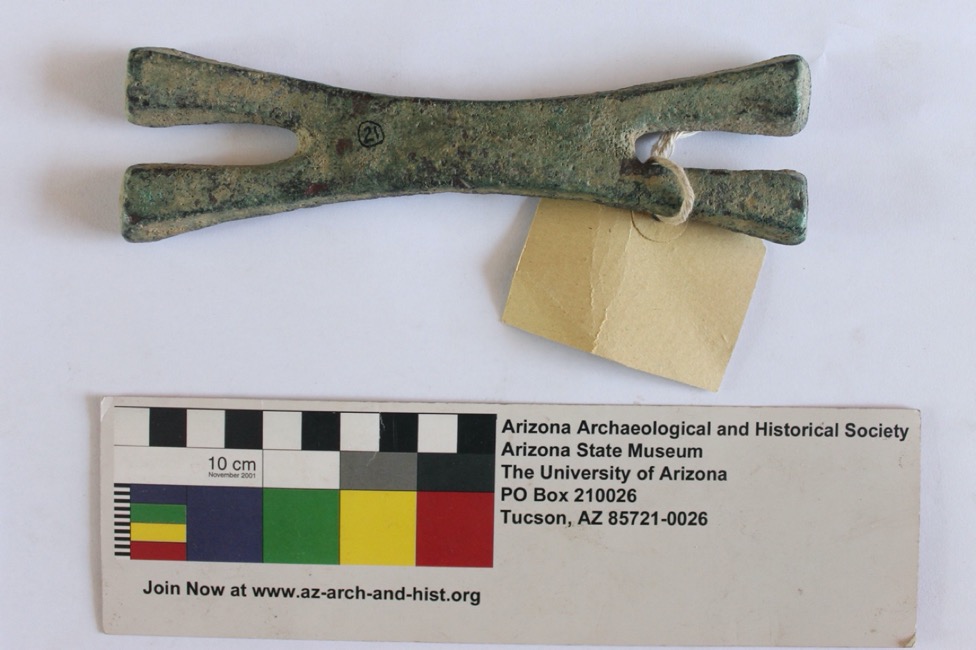


| Sample ID | Zim-ZMHS-19 |
| --- | --- |
| Ingot Type | HXR |
| Country | Zimbabwe |
| Site | Karoi area |
| Museum Site Reference | - |
| Cultural Association | - |
| Context | - |
| Housing Museum | Museum of Human Sciences, Harare |
| Museum Accession Number | QMIA 4496 |
| Reference | Swan 2007 |
| Weight (g) | 3795 |
| Length (cm) | 34 |


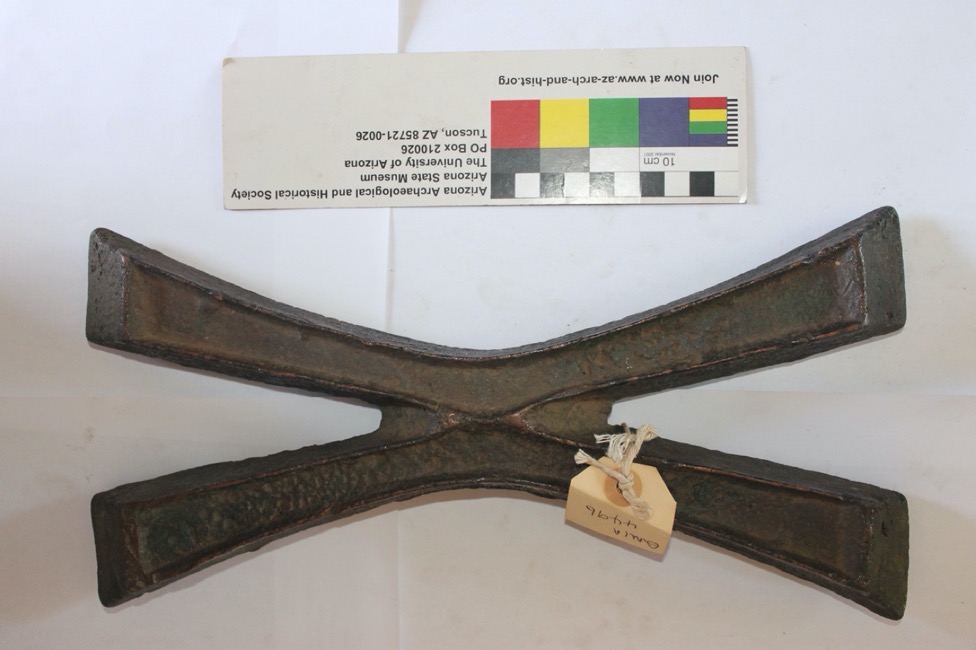


| Sample ID | Zim-ZMHS-21 |
| --- | --- |
| Ingot Type | HXR |
| Country | Zimbabwe |
| Site | Gil Gil Mine |
| Museum Site Reference | 1629 CA 1 |
| Cultural Association | - |
| Context | - |
| Housing Museum | Museum of Human Sciences, Harare |
| Museum Accession Number | QMIA 4478 |
| Reference | Swan 2007 |
| Weight (g) | - (Partial Ingot) |
| Length (cm) | Estimated 38cm (Partial Ingot) |


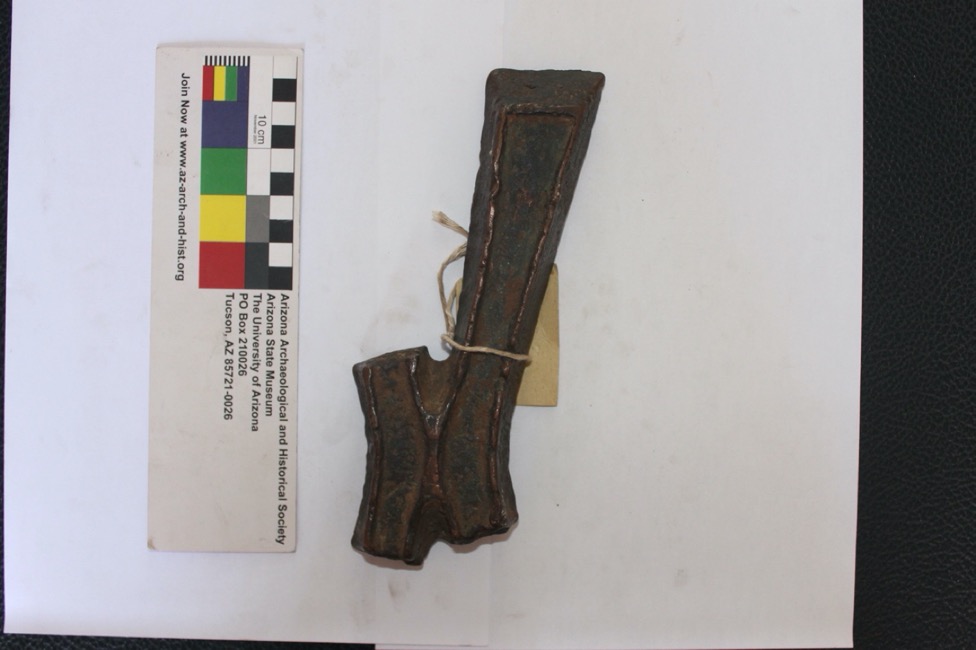


| Sample ID | Zim-ZMHS-22 |
| --- | --- |
| Ingot Type | HXR |
| Country | Zimbabwe |
| Site | Easter Parade Farm |
| Museum Site Reference | 1629 DA 24 |
| Cultural Association | - |
| Context | - |
| Housing Museum | Museum of Human Sciences, Harare |
| Museum Accession Number | - |
| Reference | - |
| Weight (g) | - (Partial Ingot) |
| Length (cm) | - (Partial Ingot) |


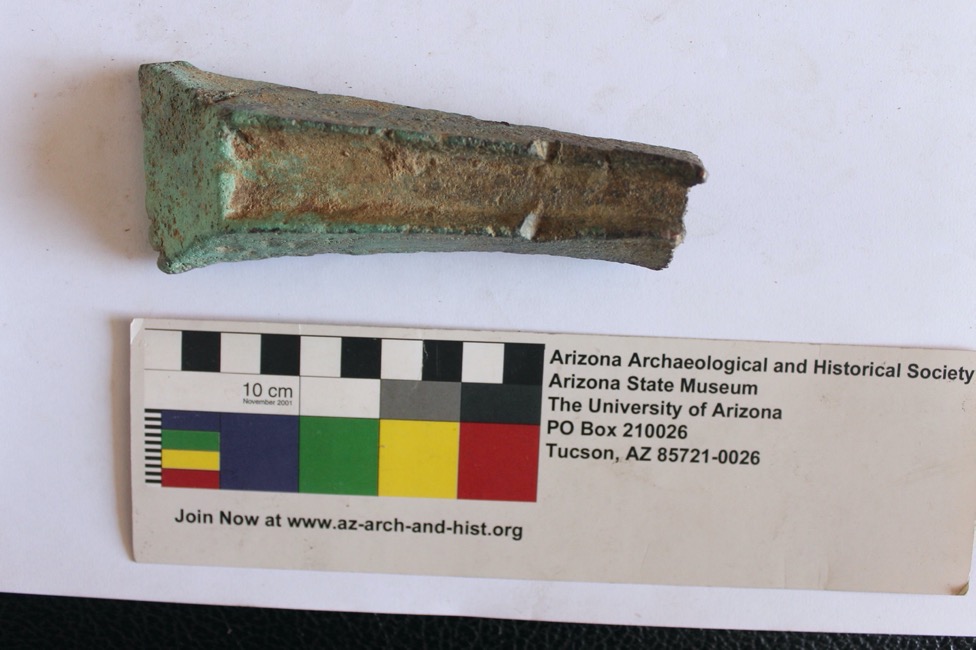


| Sample ID | Zim-ZMHS-23 |
| --- | --- |
| Ingot Type | HIH |
| Country | Zimbabwe |
| Site | Mwami |
| Museum Site Reference | 1629 DB 10 |
| Cultural Association | - |
| Context | - |
| Housing Museum | Museum of Human Sciences, Harare |
| Museum Accession Number | - |
| Reference | Swan 2007 |
| Weight (g) | 549 |
| Length (cm) | 17.2 |


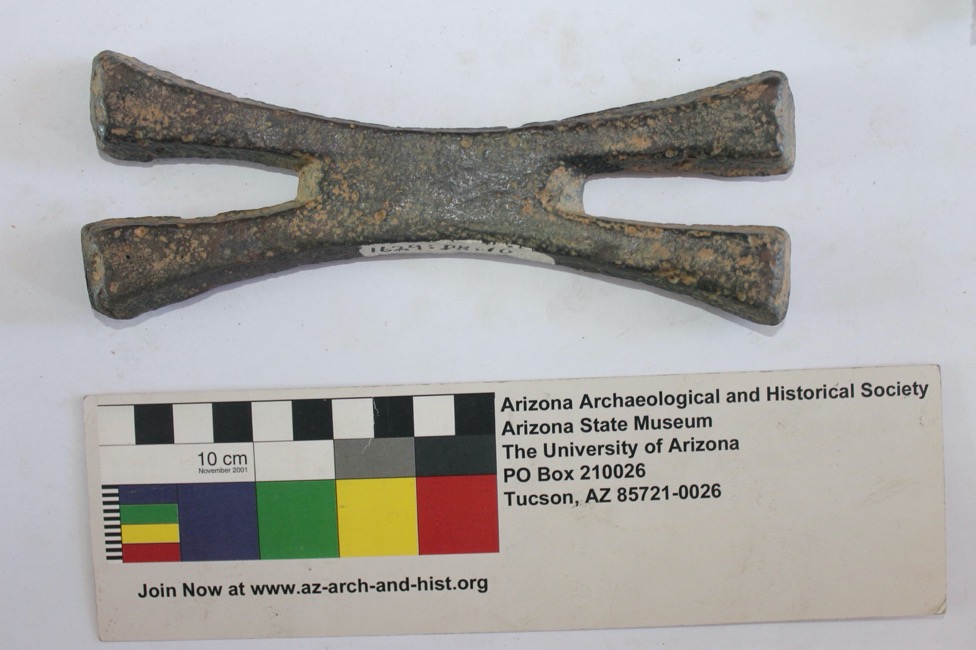


| Sample ID | Zim-ZMHS-24 |
| --- | --- |
| Ingot Type | HIH |
| Country | Zimbabwe |
| Site | Beatrice area |
| Museum Site Reference | 1830 BD 12 |
| Cultural Association | - |
| Context | - |
| Housing Museum | Museum of Human Sciences, Harare |
| Museum Accession Number | QMIA 4484 |
| Reference | Swan 2007 |
| Weight (g) | 348 |
| Length (cm) | 17.2 |


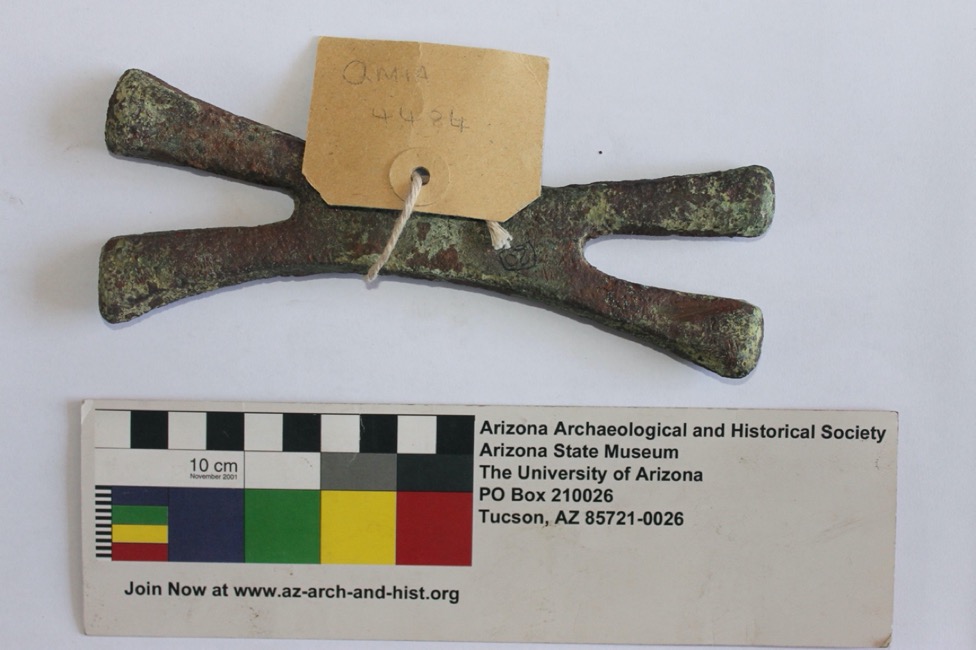


| Sample ID | Zim-Ship-1 |
| --- | --- |
| Ingot Type | HIH |
| Country | Zimbabwe |
| Site | Shipton Farm |
| Museum Site Reference | 1730 CB 8 |
| Cultural Association | - |
| Context | - |
| Housing Museum | Museum of Human Sciences, Harare |
| Museum Accession Number | - |
| Reference | - |
| Weight (g) | - |
| Length (cm) | 20 |


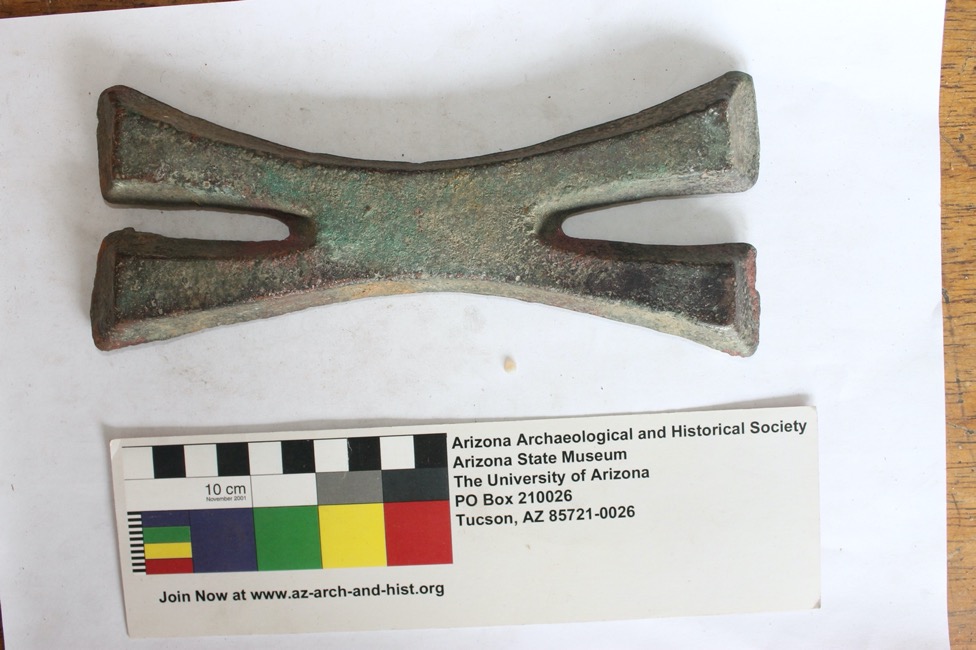


| Sample ID | Zam-II-7 |
| --- | --- |
| Ingot Type | HXR |
| Country | Zambia |
| Site | Ingombe Ilede |
| Museum Site Reference | - |
| Cultural Association | Ingombe Ilede |
| Context | Burial 8 |
| Housing Museum | Livingstone Museum, Livingstone |
| Museum Accession Number | - |
| Reference | Fagan et al. 1969 |
| Weight (g) | 4080 |
| Length (cm) | 31 |


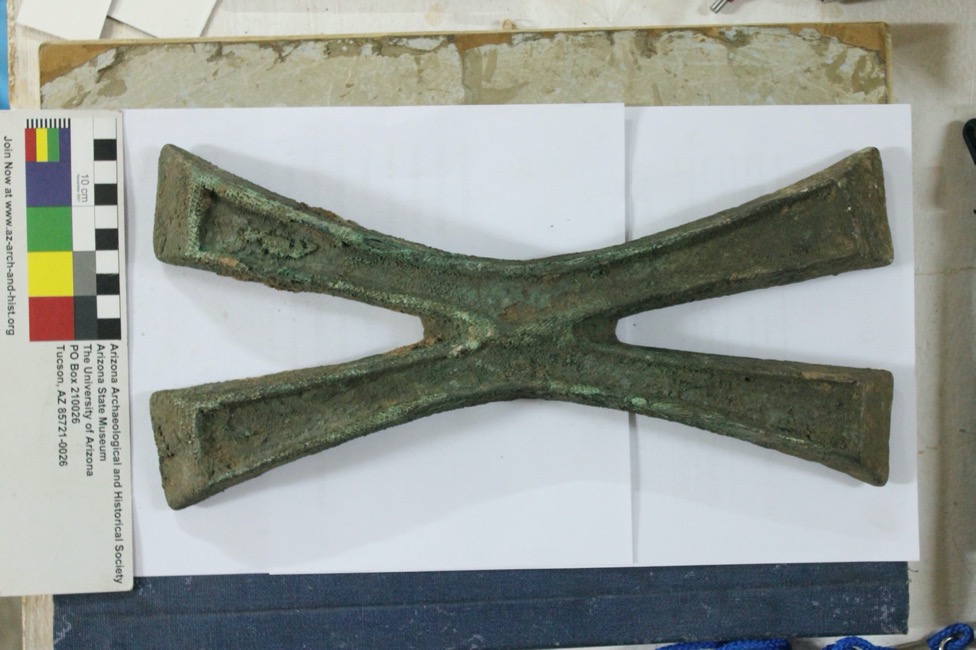


| Sample ID | Zam-II-14 |
| --- | --- |
| Ingot Type | HXR |
| Country | Zambia |
| Site | Ingombe Ilede |
| Museum Site Reference | - |
| Cultural Association | Ingombe Ilede |
| Context | Burial 8 |
| Housing Museum | Livingstone Museum, Livingstone |
| Museum Accession Number | - |
| Reference | Fagan et al. 1969 |
| Weight (g) | 3970 |
| Length (cm) | 36 |


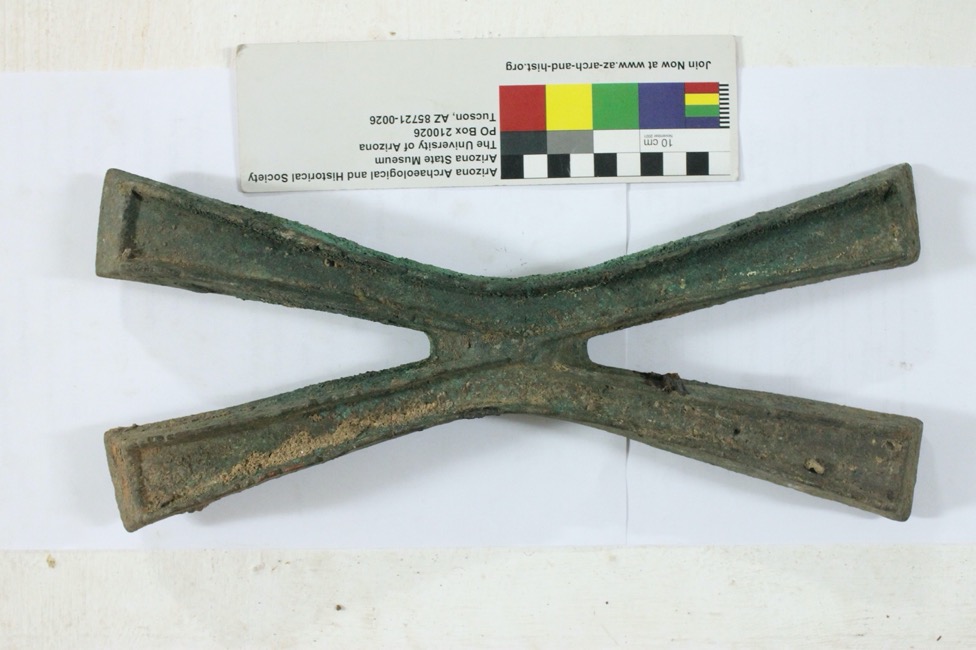


| Sample ID | Zam-II-15 |
| --- | --- |
| Ingot Type | HXR |
| Country | Zambia |
| Site | Ingombe Ilede |
| Museum Site Reference | - |
| Cultural Association | Ingombe Ilede |
| Context | Burial 2 |
| Housing Museum | Livingstone Museum, Livingstone |
| Museum Accession Number | - |
| Reference | Fagan et al. 1969 |
| Weight (g) | 2310.5 |
| Length (cm) | 31.5 |


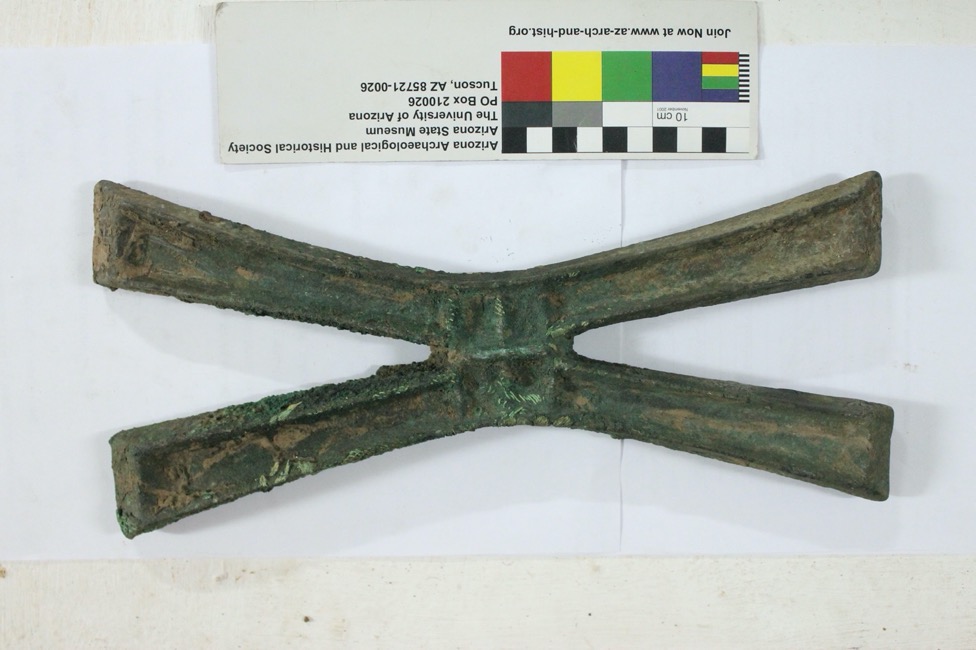


| Sample ID | Zam-Kuma-1 |
| --- | --- |
| Ingot Type | Ia (Rectangular) |
| Country | Zambia |
| Site | Kumadzulo |
| Museum Site Reference | - |
| Cultural Association | Early Iron Age |
| Context | Unknown (label reads K20 V.C.5 IV) |
| Housing Museum | Livingstone Museum, Livingstone |
| Museum Accession Number | - |
| Reference | Vogel 1971 |
| Weight (g) | - (Partial Ingot) |
| Length (cm) | - (Partial Ingot) |


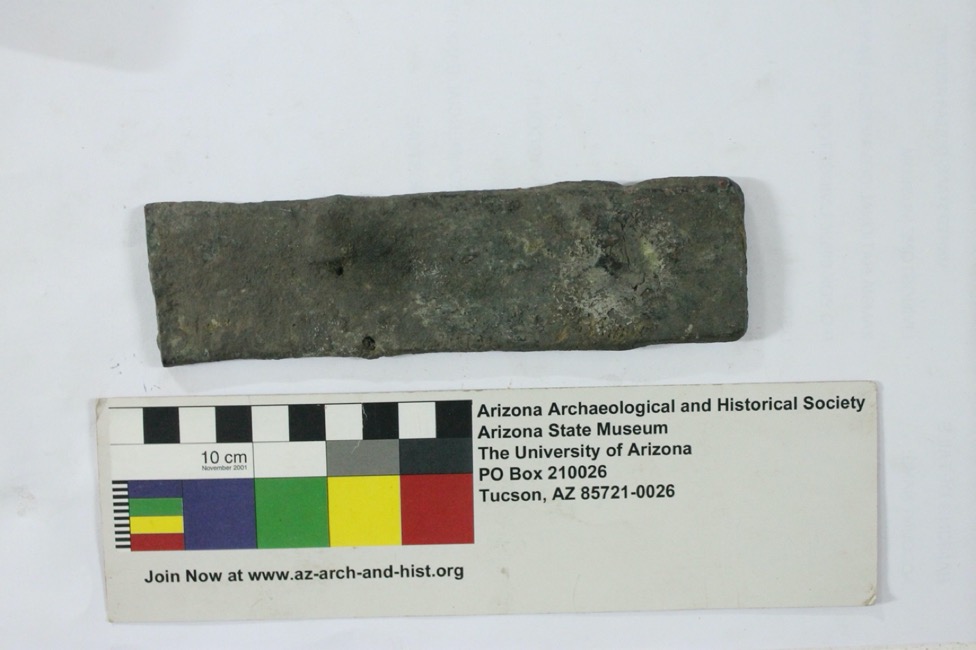


| Sample ID | Zam-Kamu-1 |
| --- | --- |
| Ingot Type | Ia (Fishtail) |
| Country | Zambia |
| Site | Kamusongolwa |
| Museum Site Reference | - |
| Cultural Association | Early Iron Age |
| Context | Locality B, 10” below surface |
| Housing Museum | Livingstone Museum, Livingstone |
| Museum Accession Number | - |
| Reference | Daniels 1967 |
| Weight (g) | - |
| Length (cm) | 30 |


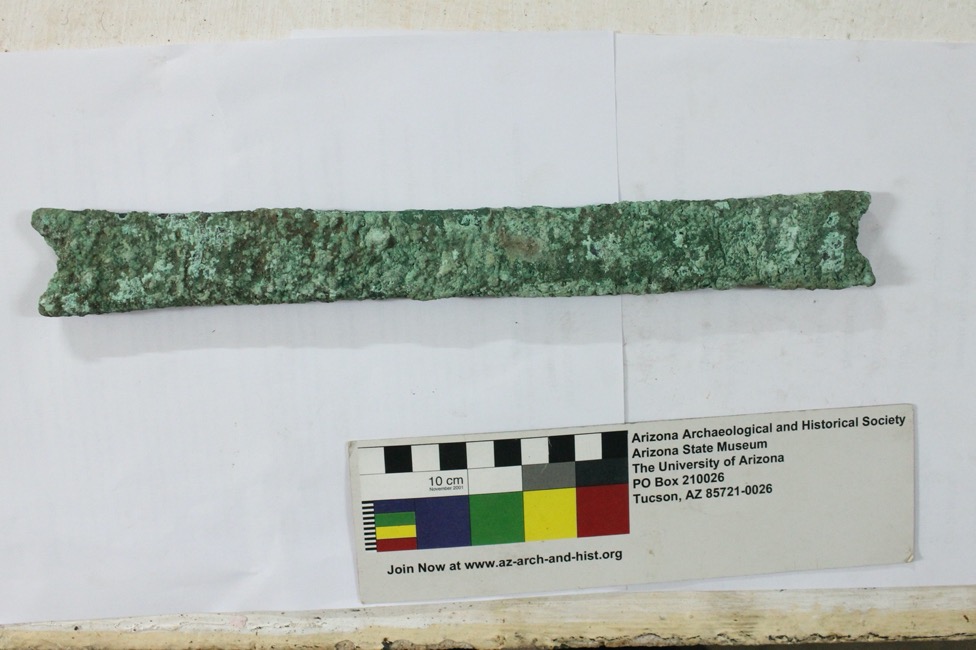


| Sample ID | Zam-Kan-19 |
| --- | --- |
| Ingot Type | Experimental X |
| Country | Zambia |
| Site | Kansanshi |
| Museum Site Reference | - |
| Cultural Association | Modern |
| Context | Modern X-shaped ingot cast from Kansanshi copper ore |
| Housing Museum | - |
| Museum Accession Number | - |
| Reference | - |
| Weight (g) | - |
| Length (cm) | - |


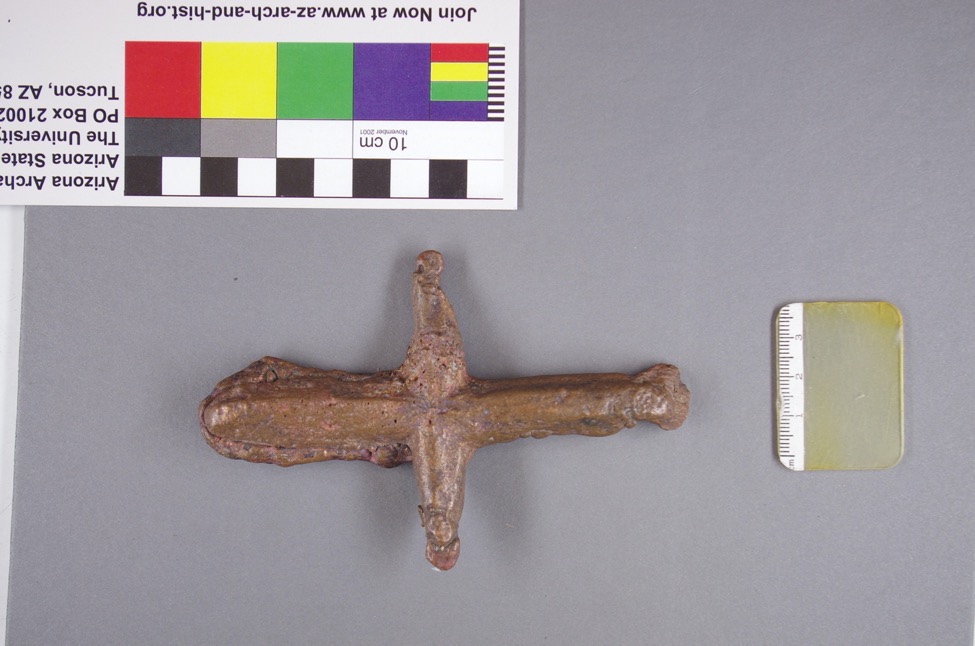


| Sample ID | Zam-Luano-4 |
| --- | --- |
| Ingot Type | Ia (Fishtail) |
| Country | Zambia |
| Site | Luano main site |
| Museum Site Reference | - |
| Cultural Association | Early Iron Age |
| Context | - |
| Housing Museum | Livingstone Museum, Livingstone |
| Museum Accession Number | - |
| Reference | Bisson 2000 |
| Weight (g) | - (Partial Ingot) |
| Length (cm) | 14.4 (Partial Ingot) |


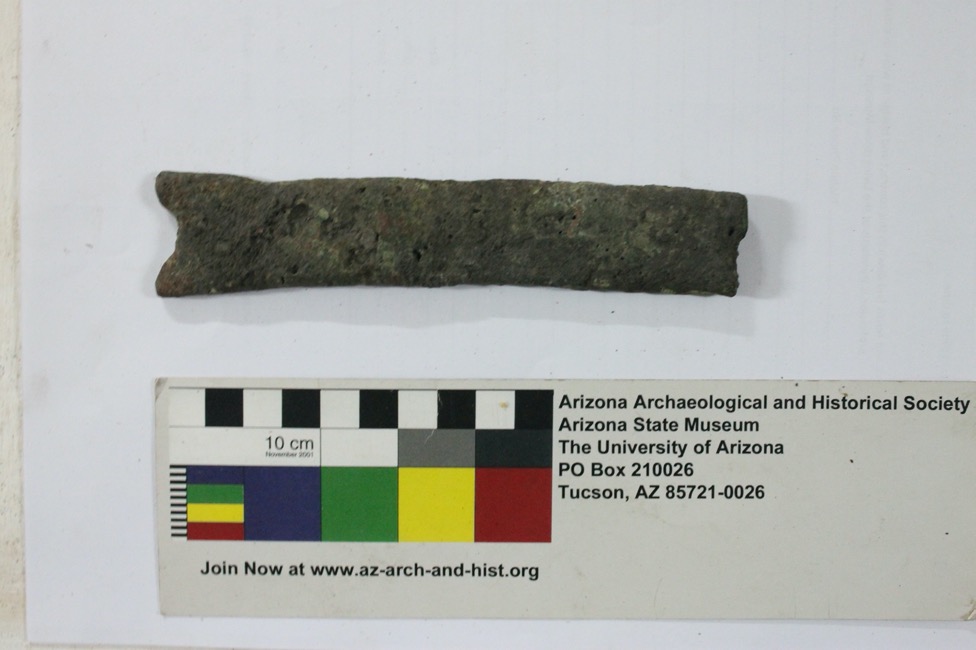

Supplement: S2 Appendix — (DOCX) [file pone.0282660.s002.docx]
